# *Artemisia annua*-derived extracellular vesicles reprogram breast tumor immune microenvironment via altering macrophage polarization and synergizing recruitment of T lymphocytes

**DOI:** 10.1186/s13020-025-01210-1

**Published:** 2025-10-01

**Authors:** Yun Wang, Lin Meng, Sicheng Su, Yu Zhao, Xiaoxian Hu, Xiaoqing Xu, Chao Han, Jianguang Luo, Zhongrui Li

**Affiliations:** 1 Department of Rehabilitation, College of Acupuncture and Moxibustion and Massage Health Preservation and Rehabilitation, Nanjing University of Chinese Medicine, Nanjing, 210023 People’s Republic of China; 2 Basic Medical Research Innovation Center for Anti-Cancer Drugs, State Key Laboratory of Natural Medicines, Jiangsu Key Laboratory of Bioactive Natural Product Research, School of Traditional Chinese Pharmacy, China Pharmaceutical University, Nanjing, 211198 China

**Keywords:** Breast cancer, Extracellular vesicles, *Artemisia annua*, Macrophage polarization, T lymphocyte infiltration

## Abstract

**Background:**

The immunosuppressive microenvironment and limited immune cell infiltration into the tumor bed contribute to the proliferation, metastasis, and invasion of breast cancer (BC) cells. Reprogramming the tumor immune microenvironment has emerged as a promising therapeutic target for BC, but remains challenging in clinical practice. *Artemisia annua*, a medicinal plant, has shown immune-enhancing and anti-tumor activities, although its potential therapeutic applications in BC remain underexplored.

**Methods:**

Extracellular vesicles (EVs) were isolated from fresh *Artemisia annua* using gradient centrifugation and characterized by transmission electron microscopy (TEM), nanoparticle tracking analysis (NTA), Zetasizer Nano ZS90, ultra-high-performance liquid chromatography-mass spectrometry (UHPLC-MS/MS), and high-performance liquid chromatography (HPLC). Single-cell RNA sequencing (scRNA-seq) analysis was performed to investigate the mechanism of AEVs on tumor growth in vivo and mRNA sequencing (mRNA-seq) was employed to further explore the mechanism of AEVs-induced polarization shift from M2-like to M1-like macrophages. In vitro and in vivo assays were conducted to assess the polarization of macrophages and recruitment of T lymphocytes into the tumor bed.

**Results:**

AEVs were successfully isolated and characterized. In vivo, AEVs inhibited tumor growth by shifting macrophage polarization from the M2-like to the M1-like phenotype, and synergistically enhancing the recruitment of CD8^+^ and CD4^+^ T cells into the tumor microenvironment. AEVs activated the NF-κB signaling pathway while inhibiting the PPARγ pathway, thereby promoting the M1-like polarization of macrophages. Polarized M1-like macrophages secreted chemokines (CCL5 and CCL3), which facilitated T lymphocyte infiltration into the tumor bed. Notably, AEVs reshaped the BC immune microenvironment without inducing systemic toxicity.

**Conclusions:**

AEVs from *Artemisia annua* efficiently reprogrammed the immune microenvironment of breast cancer by inducing macrophage polarization and enhancing T lymphocyte infiltration. This study lays the foundation for using AEVs as a potential immunotherapy for BC and highlights the medicinal value of *Artemisia annua* in cancer treatment.

**Supplementary Information:**

The online version contains supplementary material available at 10.1186/s13020-025-01210-1.

## Introduction

As the leading cancer type by incidence, breast cancer (BC) is anticipated to represent 32% of all new cancer diagnoses in women worldwide by 2025, highlighting its enduring significance as a women's health priority [1, 2]. This substantial disease impact continues unabated notwithstanding the availability of comprehensive treatment arsenals including ablative surgery, antineoplastic drugs, and radiotherapeutic interventions [3, 4]. One of the reasons for treatment failure may be the inability to establish an efficient tumor-specific immune response in the host [5]. The tumor microenvironment (TME) plays a critical role in the development and progression of cancer, which is composed of various cell types (such as tumor cells, immune cells, endothelial cells, and fibroblasts) and extracellular components (such as various cytokines, growth factors, chemokines, and the extracellular matrix) [6, 7]. Recently, an increasing number of studies [5, 8, 9] have confirmed that the BC microenvironment, including suppressive immune cells and tumor-promoting cytokines, inhibits effective antitumor immunity and promotes the occurrence and progression of BC. Reprogramming the tumor immune microenvironment to enhance antitumor immunity is regarded as a promising therapeutic approach for the clinical treatment of BC, but this approach remains highly challenging.

Tumor-associated macrophages (TAMs) are a major component of the TME, and their polarization is related to tumor immune escape, growth, and metastasis [10, 11]. M1-like macrophages are capable of proinflammatory responses and antitumor effects and can produce related factors such as interleukin-6 (IL-6), IL-12 and tumor necrosis factor α (TNF-α). In contrast, M2-like macrophages can secrete arginase-1 (Arg-1) and IL-10 to exert anti-inflammatory and protumor effects. Therefore, the exhaustion of M2-like macrophages or the induction of M1-like polarization can reprogram the TME, which has been used to treat cancer [12, 13]. In addition to TAMs, T lymphocytes play crucial roles in adaptive immune responses to tumors. For example, CD8^+^ T cells are key cytotoxic lymphocytes that are responsible for inhibiting tumor proliferation by directly recognizing and killing tumor cells via intracellular antigens [14, 15]. Moreover, CD4^+^ T cells may also exert antitumor effects by assisting CD8^+^ T cells or, in some cases, directly recognizing antigens presented on the surface of tumor cells eventually followed by secretion of type 1 cytokines (TNF-α or IFN-γ) or direct cancer killing [16, 17]. Furthermore, clinical data suggest that a low level of T lymphocyte infiltrations in BC lesions is a passive prognostic factor [18]. Thus, reprogramming the tumor immune microenvironment by altering macrophage polarization and synergizing with the recruitment of T lymphocytes is a promising treatment for BC.

*Artemisia annua* L. is a commonly used traditional Chinese medicine, and has been used for the treatment of various diseases since ancient times, including intermittent fevers due to malaria, bone steaming and heat/fever arising from exhaustion [19]. The well-known antimalarial drug artemisinin was isolated from this plant for the first time. Modern pharmacological studies have shown that *A. annua* possesses immune-enhancing, antitumor and anti-inflammatory effects, and has been employed for the treatment of BC, non-small cell lung cancer, leukemia, prostate cancer, colon cancer, renal cell carcinoma and hepatoma [20, 21]. Fresh plant-derived extracellular vesicles (EVs) are small lipid-based membrane-bound entities that contain various proteins, polysaccharides, miRNAs, and other active substances [22]. Plant-derived EVs can serve as extracellular messengers to regulate intercellular communication. An increasing number of studies have demonstrated the potential remodeling of the TME plant-derived EVs in multiple cancer models [23]. For example, ginseng-based EVs can potentiate PD-1 mAb therapy by recruiting CD8^+^ T cells into the cold TME [24]. After oral administration of garlic-derived EVs, the translocation of γδ T cells and IFN-γ from the gut to the TME remodeled the tumor immune microenvironment and synergized with anti-PD-L1, inducing antitumor immunity [25]. Thus, plant-derived EVs constitute an alternative approach for cancer immunotherapy.

Here, we successfully prepared EVs from fresh *A. annua* by using gradient centrifugation. *A. annua*-based EVs (AEVs) contain mainly proteins, lipids, amino acids, and small molecular compounds. Interestingly, artemisinin was present only in AEVs from the juice of *A. annua*. Single-cell RNA-sequencing (scRNA-seq) analysis revealed that AEVs inhibited the growth of tumors by altering macrophage polarization and synergizing with the recruitment of T lymphocytes in vivo. AEVs promoted the polarization of macrophages from the M2-like to the M1-like phenotype by activating NF-κB and inhibiting the PPARγ signaling pathway. Moreover, polarized M1-like macrophages secreted chemokines (CCL5 and CCL3) to promote T lymphocyte infiltration. Our work shows for the first time that AEVs exert an immunomodulatory effect to inhibit BC growth and provides the basis for BC immunotherapy.

## Materials and methods

### Preparation of AEVs

The fresh herb of *A. annua* was purchased from Bozhou local herbal market, Anhui, China, and identified by Professor Minjian Qin from the Research Department of Pharmacognosy, China Pharmaceutical University. The fresh ground parts (2 kg) were first washed with deionized H_2_O three times, and then placed in a slow juicer to obtain juice. Next, the juice was sequentially centrifuged at 200 × *g* for 10 min, 2000 × *g* for 20 min, and 10,000 × *g* for 30 min to remove large particles and fibres. The supernatant was ultracentrifuged at 100,000 × *g* for 1 h to collect precipitate. The obtained precipitate was resuspended in phosphate buffer saline (PBS) and transferred to a gradient sucrose solution (15, 30, 45 and 60%). After another ultracentrifugation (150,000 × g, 1 h), the band of 45% sucrose layer was obtained. Finally, the AEVs were collected after removing sucrose. The obtained AEVs were resuspended in PBS for use freshly or stored at − 80 °C until further use.

### Characterization of AEVs

Morphological characteristics of AEVs were determined by HT7700 transmission electron microscopy (Hitachi, Tokyo, Japan). The particle size was characterized by a nanoparticle tracking analysis system (ZetaView, Meerbusch, Germany), and the zeta potential was characterized by Zetasizer Nano ZS90 (Malvern, Worcestershire, UK). Untargeted metabolomics of AEVs was analyzed by UHPLC-MS/MS, which was entrusted to Applied Protein Technology, Shanghai, China. The extracts, AEVs, and artemisinin standard (Aladdin Reagent, Shanghai, China) were measured using an Agilent 1100 HPLC with column (250 × 4.6 mm, 5 μm; Shimadzu, Kyoto, Japan). The HPLC conditions were as follows: mobile phase: acetonitrile:water (60:40, v/v); column temperature: 25 °C; flow rate: 1 mL min^−1^; wavelength, 210 nm. The release of artemisinin in vitro was determined by dialysis under pH 5.5 and pH 7.4 of PBS, respectively. AEVs (2 mL) were soaked in PBS (35 mL) containing 0.1% tween 80. The release of artemisinin was analyzed by using HPLC.

### Animal study

All animal experiments were approved by the Animal Ethical Committee of China Pharmaceutical University, and the handling procedures were performed on the basis of the National Institutes of Health of Experimental Animals.

### *Biodistribution and biosafety evaluation *in vivo

Five-week-old BALB/c female mice (GemPharmatech, Nanjing, China) were intravenously injected with DiD-labeled AEVs or saline. Then, the mice were imaged (Ex: 644 nm, Em: 663 nm) at 1, 4, 12 and 24 h post-injection by the IVIS Spectrum In Vivo Imaging System (Caliper, Massachusetts, USA). At 24 h, the mice were euthanized and sacrificed, and the main organs were collected and imaged. The blood samples from mice were obtained for biochemical analyses, and the major organs were sliced for pathological section analysis.

### Acute toxicity test

A cohort of five-week-old female ICR mice (Gempharmatech Co., Ltd., Jiangsu, China) was randomly allocated into five experimental groups (n = 10 per group). Each group received intravenously injections of increasing doses of AEVs (50, 100, 200, 400, and 800 mg/kg, respectively). Following administration, the animals were monitored for 14 days to assess clinical signs, including alterations in fur condition, behavioral responses, body weight fluctuations, and survival outcomes. To evaluate potential organ toxicity, three mice from the highest-dose group (800 mg/kg) were euthanized for histopathological examination. Tissues from major organs—including the heart, liver, spleen, lung, and kidney—were harvested, processed, and subjected to microscopic analysis to identify any pathological changes.

### Cell culture

RAW 264.7, and THP-1 cells were purchased from the Cell Bank of Shanghai Institute of Biochemistry and Cell Biology, Shanghai, China. 4T1, EMT-6, and C127 cells were obtained from Procell Life Science & Technology, Wuhan, China. RAW 264.7 and C127 cells were cultured in DMEM supplemented with 10% fetal bovine serum and 1% penicillin/streptomycin at 37 °C and 5% CO_2_. THP-1, 4T1, and EMT-6 cells were cultured in RPMI-1640 culture medium.

### scRNA-seq process

Five-week-old BALB/c female mice were selected and injected with EMT-6 cells (1.0 × 10^7^ cells per mouse) into the right lower limb region. When the tumors reached a size of 100 mm^3^, the mice were randomly divided into two groups (5 mice/group). The mice were intratumorally treated with AEVs (200 μg/100 μL/mouse) or saline every two days. After 15 days of treatment, tumor tissues were obtained and digested to single-cell suspensions by using a tumor dissociation kit (Miltenyi Biotec, Bergisch Gladbach, Germany). Single-cell suspensions were loaded on the 10 × Genomics single-cell analysis system, which were commissioned for OE Biotech, Shanghai, China. Barcoded gel beads and cells were enclosed in the droplet by means of microfluidic technology. Cells mRNA was connected with the beads to generate GEMs for reverse transcription.

### Quality control and analysis of scRNA-seq data

The scRNA-seq data were analyzed by with Cell Ranger software (10 × Genomics). Before downstream analysis, low-quality cells (number of genes < 200, UMIs < 1000, log10 Genes Per UMI < 0.7, percentage of mitochondrial genes < 5%, and red blood cell gene expression < 5%) were deleted. Principal Components Analysis and Unified Manifold Approximation and Projection were used to obtain the optimal cell cluster. The difference between the specified cell population and all other cell populations was tested by using Presto analysis to obtain marker genes. Finally, the cell types were identified by analyzing the differentially expressed genes and known gene signatures by means of the SingleR package. The complete analytical workflow is illustrated in Supplementary Fig. S4.

### Cellular uptake of M2-like macrophages

RAW264.7 and THP-1 cells were achieved M2-like polarization by treatment with human/mouse 20 ng/mL IL-4 and 20 ng/mL IL-13. Cells (M2-like macrophages, EMT-6, and MCF-7) were seeded in 96-well plates (5 × 10^3^ cells per well) and cultured for 12 h. Then cells were incubated with Dil-labeled AEVs (20 μg/mL) for 12 h. After nuclei were stained by DAPI, cells were investigated by a high-content screening system (ImageXpress Micro Confocal, CA, USA) and flow cytometry (BD Biosciences, Franklin, USA).

### *Analysis of AEVs inducing M2-like macrophages polarization *in vitro

M2-like macrophages (M2-RAW264.7 and M2-THP-1) were seeded in 6-well plates (1 × 10^6^ cells per well) and cultured for 12 h. Then cells were incubated with AEVs (20 μg/mL) for 48 h. Supernatants were collected for detection of IL-6 and TNF-α by using an ELISA kit (Vazyme Biotech, Nanjing, China). Cells were labelled by monoclonal antibodies (Table S1) and analyzed by flow cytometry.

### Polarization of bone marrow-derived macrophages

Following euthanasia of mice, the animals were surface-sterilized by immersion in 75% ethanol for 5 min. Under sterile conditions in a biosafety cabinet, the tibiae and femurs were dissected using scissors and forceps, and attached muscle tissue was removed. The bone ends were trimmed, and bone marrow cells were flushed out by repeatedly rinsing the marrow cavity with DMEM using a syringe. The resulting cell suspension was centrifuged at 1000 × *g* for 5 min, and the supernatant was discarded. The pellet was gently resuspended in 4 mL of complete medium (DMEM supplemented with 10% fetal bovine serum and 1% penicillin/streptomycin) containing 10 ng/mL M-CSF and transferred to a culture dish. After 3 days of incubation, the medium was replaced with fresh complete medium containing the same concentration of M-CSF. By day 6, adherent mature macrophages were confirmed under microscopy and subsequently used for further experiments.

After maturation, bone marrow-derived macrophages were polarized. For M2 polarization, cells were treated with IL-4 (final concentration: 20 ng/mL) in the culture medium for 24 h.

### Phagocytosis assay

THP-1 cells were differentiated into adherent macrophages by treatment with 50 nM PMA for 24 h. Subsequently, a subset of RAW264.7 cells and differentiated THP-1 cells were polarized toward the M2 phenotype by culturing in complete medium supplemented with 20 ng/mL IL-4 for 24 h. Parallel cultures were polarized toward the M1 phenotype using 100 ng/mL LPS and 20 ng/mL IFN-γ. Following polarization, macrophages and 4T1 cells were harvested using 0.25% trypsin, centrifuged, and resuspended in sterile PBS for counting. CFSE (ThermoFisher, Waltham, MA, USA) and eFluor^™^ 670 (ThermoFisher, Waltham, MA, USA) were diluted 1:1000 in PBS. The dyes were added to suspensions of macrophages or tumor cells, vortexed, and incubated at 37 °C for 10 min protected from Light. The labeling reaction was quenched by adding complete medium, followed by a 5 min incubation and centrifugation to remove unbound dye. Labeled 4T1 cells were co-cultured with RAW264.7 macrophages at a 1:2 ratio (tumor cells:macrophages). After 12 h, cells were collected and analyzed by flow cytometry to determine phagocytic activity, defined as the percentage of double-positive macrophages (eFluor™ 670⁺/CFSE⁺) engulfing tumor cells.

### In vitro* anti-tumor efficacy*

M2-like macrophages (M2-RAW264.7 and M2-THP-1) were seeded in 96-well plates (3.0 × 10^3^ cells per well) and incubated with different dosage of AEVs for 48 h. The supernatants were separated and added to 96-well plates with MCF-7 or EMT-6 cells (3.0 × 10^3^ cells) for another 24 h. MTT solution (10 μL) was added to each well, and then 150  μL DMSO was added to dissolve the formazan. Each sample was measured at 570 nm using a SpectraMax Plus384 microplate reader (Molecular Devices, CA, USA). Apoptosis induction was proceeded by using an Annexin V-FITC/PI apoptosis detection kit (KeyGen BioTech, Nanjing, China) and analyzed by flow cytometry.

### In vivo* anti-tumor efficacy*

Five-week-old BALB/c female mice were selected and injected with EMT-6, C127 or 4T1 cells (1.0 × 10^6^ cells per mouse) into the right lower limb region. When the tumors reached a size of 100 mm^3^, the mice were randomly divided into three groups (5 mice/group). Then, the mice were intratumorally treated with saline, AEVs (200 μg/100 μl/mouse), and AEVs + clodronate liposome (CL) every two days. The body weight and tumor volume (length × width^2^ × 0.5) were measured. After administration for 14 days, the tumors and main organs were harvested for pathological section analysis. Fresh tumor samples were minced to obtain single cell suspensions, then cells were sorted by different monoclonal antibody. Flow cytometry was used to analyze macrophages and infiltrating T cells.

### mRNA-seq

mRNA-seq analysis was supported by Gene Denovo (Guangzhou, China). M2-like RAW264.7 cells were incubated with or without AEVs (20 μg/mL) for 48 h. Then, cells were collected and added with 2 mL of RNA extraction reagent. Triplicate samples were required for each group. The significant probe sets were filtered for measurement by using a fold-change > 1.5, *p* < 0.05 (Student’s *t*-test) and false discovery rate < 0.05. The differentially expressed genes and signal path were enriched in GO and KEGG. Quality control was also proceeded by Gene Denovo.

### Western blotting

M2-like RAW264.7 cells were cultured in 6-well plates (1 × 10^6^ cells per well) for 12 h, and treated with drugs for different times. Then, cells were collected to extract total protein. Equal quantities of protein from different groups were separated by sodium dodecyl sulfate (10%) polyacrylamide gel electrophoresis. The proteins were transferred to a polyvinylidene fluoride membrane. The samples were blotted with primary antibodies (Table S1) for 24 h at 4 °C. Then, the secondary antibody was incubated with the sample for 2 h at 37 °C. The samples were detected by a ChemiDOC^™^ XRS + system (Bio-Rad Laboratories, Hercules, CA, USA).

### Quantitative real-time polymerase chain reaction (qRT-PCR)

Total RNA of M2-like RAW264.7 cells was extracted using Trizol regent (Vazyme Biotech, Nanjing, China), and then cDNA was reverse-transcribed using HiScript II Q RT SuperMix (Vazyme Biotech, Nanjing, China). The sequences of primers are listed in Table S2. Then, Light Cycler 480 II Real-Time PCR System (Roche, Switzerland) was used to analyze qRT-PCR through using SYBR Green PCR Master Mix (Vazyme Biotech, Nanjing, China). GAPDH was used as control to normalize the mRNA expression level.

### Double luciferase reporter gene assay

PPARγ report plasmid was constructed by using pGL3-Basic vector. The core region of PPARγ gene promoter was amplified and ligated into the prepared vector. After being amplified by *E. coli* DH5*α*, the plasmids were transfected into the HEK 293 T cells. Then, cells were treated with different concentrations of AEVs or PBS for 24 h. The luminescence intensity of cells was detected by a Double-Luciferase Reporter Assay Kit (Vazyme Biotech, Nanjing, China). In addition to the PPARγ gene promoter, three STAT6 binding sites of PPARγ promoter were also mutated. The relative transcriptional activity was calculated by the RLU firefly luciferase/RLU renilla luciferase. The sequences of primers used in double luciferase reporter gene assay were listed in Table S3.

### *Chemotaxis assay *in vitro* and *in vivo

4T1 tumor-bearing mice were intratumorally treated with AEVs (200 μg/100 μL/mouse) or saline on day 10. The DiO-labeled CD8^+^ T cells (5 × 10^5^ cells/100 μL/mouse) were intravenously injected into the mice on day 11. Mice were sacrificed on day 13. Tumor tissues were collected and analyzed the chemotactic DiO-CD8^+^ T lymphocytes by using immunofluorescent. For CD8^+^ T lymphocytes migration in vitro, conditioned media (150 μL/well) of AEVs-treated macrophage supernatant were added in the bottom of the Transwell migration chamber (Corning Life Sciences, MA, USA). The conditioned media also contained 1 μg/mL anti-CCL5 and 1 μg/mL anti-CCL3. Then, CD8^+^ T cells (3 × 10^4^) were added to the top chamber in the DMEM culture media. Migration was calculated by enumerating the number of migrated cells in the bottom of chamber after 24 h.

### *Neutralization assay of CCL5 and CCL3 *in vivo

4T1 tumor-bearing mice were randomly divided into five groups (5 mice/group), which were treated with saline, AEVs, AEVs + αCCL5, AEVs + αCCL3, and AEVs + αCCL5 + αCCL3, respectively. The mice were intratumorally injected with AEVs (200 μg/100 μL/mouse) or saline on day 10, 12, 14, 17, and 20, respectively. Furthermore, the mice were also intratumorally treated with αCCL5 (50 μg/100 μL/mouse) or αCCL3 (50 μg/100 μL/mouse) on day 11, 13, 15, 18, and 21, respectively. The body weight and tumor volume (length × width^2^ × 0.5) were measured. Mice were sacrificed on day 24 and the tumor weight was calculated by an electronic weighing machine.

### Statistical analysis

All experiments were repeated at least in triplicate and data were expressed as the mean ± standard deviation (SD). Results were analyzed by Student’s *t*-test and one-way analysis of variance by using GraphPad Prism software. Statistically significant was defined as ^*^p < 0.05, ^**^p < 0.01, ^***^p < 0.001, and ^****^p < 0.0001.

## Results

### Isolation, characterization and biosafety evaluation of AEVs

**Fig. 1:**
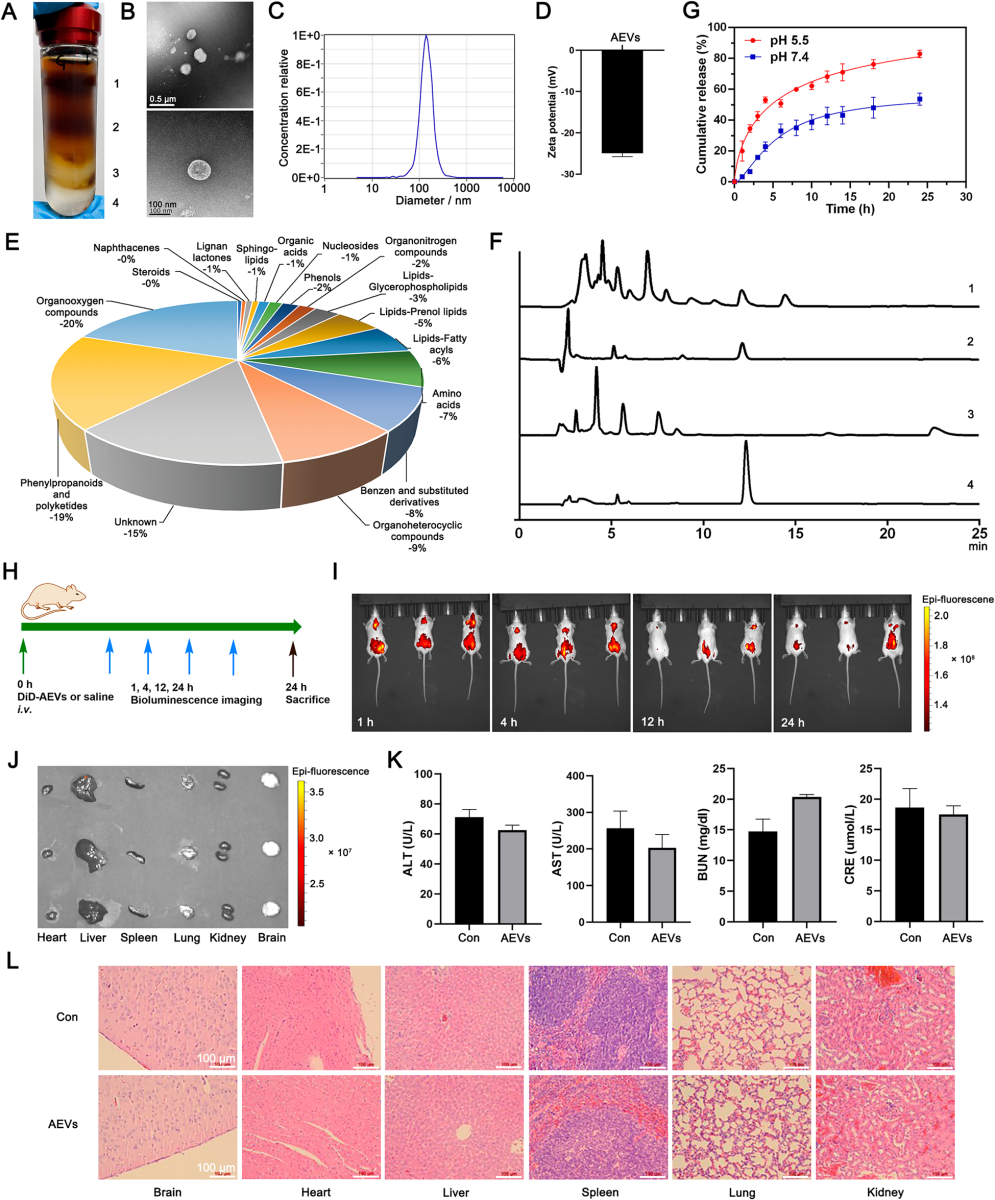
Characterization and biosafety evaluation of AEVs. **A** Ultracentrifugation of *A. annua*-based juice by sucrose density gradient. 1: 15% sucrose band; 2: 30% sucrose band; 3: 45% sucrose band; 4: 60% sucrose band. **B** TEM images of AEVs (**C**) Size distribution of AEVs in aqueous solution. **D** Zeta-potentials of AEVs. **E** Pie chart of the composition of AEVs by the untargeted metabolomics analysis. **F** HPLC chromatograms. 1: *A. annua*-based juice; 2: AEVs; 3: *A. annua*-based juice after ultracentrifugation; 4: Artemisinin standard. **G** In vitro artemisinin release curve from AEVs. Data were mean ± SD (n = 3). **H** Time schedule for drug treatment and imaging in BALB/c mice. **I** In vivo fluorescence images of mice after intravenous injection of DiD-AEVs. **J** Fluorescence images of organs harvested at 24 h post-injection. **K** Blood biochemistry tests of BUN (blood urea nitrogen), CRE (creatinine), ALT (alanine aminotransferase), and AST (aspartate aminotransferase) after treatment of AEVs. Data were mean ± SD (n = 3). **L** H&E staining of the main organs of mice after treatment. Scale bars = 100 μm

To obtain AEVs from *A. annua* (Supplementary Fig. S1), the fresh ground parts of *A. annua* were first crushed and juiced. Then, the vesicle populations of the juice were enriched using differential ultracentrifugation to remove the cells, cellular debris and large organelles from the juice. Following sucrose gradient ultracentrifugation, the 45% sucrose fraction (Fig. 1A) demonstrated predominant AEV enrichment, while virtually no vesicle content was detected in the 15%, 30% and 60% sucrose layers as evidenced by HPLC (Supplementary Fig. S2). Transmission electron microscopy (TEM) revealed that AEVs exhibited a uniform vesicular morphology (Fig. 1B). Comprehensive physicochemical characterization demonstrated excellent monodispersity (PDI = 0.0600 ± 0.002, Supplementary Fig. S3) with a hydrodynamic diameter of 137.2 nm (Fig. 1C) and a surface charge of −26.7 mV (Fig. 1D), indicating stable nanoparticle formation. Subsequent compositional analysis was performed using liquid chromatography-mass spectrometry (LC–MS/MS) to identify and quantify their molecular constituents. Untargeted metabolomics analysis revealed that AEVs contained mainly organooxygen compounds (− 20%), phenylpropanoids and polyketides (− 19%), lipids (− 14%), organoheterocyclic compounds (− 9%), benzene and substituted derivatives (− 8%), and amino acids (− 7%) (Fig. 1E). Moreover, the AEVs were stable in PBS and cell culture medium (Supplementary Fig. S4A); no change in particle size (Supplementary Fig. S4B) was observed over 7 days. The antimalarial drug artemisinin is derived from *A. annua*. Therefore, we studied the distribution of artemisinin in AEVs. Interestingly, artemisinin was distributed in the AEVs, whereas the juices of *A. annua* without AEVs contained no artemisinin (Fig. 1F). Further analysis revealed that other bioactive compounds—including scopoletin, chlorogenic acid, artemisinic acid, and apigenin—were absent in AEVs, suggesting a preferential packaging mechanism for artemisinin over these metabolites (Supplementary Fig. S5). Moreover, the drug loading and release properties of the AEVs were investigated. Artemisinin, as a template molecule, can be loaded into AEVs. The standard curve of artemisinin was calculated to determine the encapsulation efficiency (EE) values, which had a linear Range of 0.08–10.00 mM (Supplementary Fig. S6A). When the weight ratio of artemisinin to AEVs was 1:1, the optimum EE value was 53.9% (Supplementary Fig. S6B). The in vitro drug release profile is displayed in Fig. 1G, and the accumulative release of artemisinin from the AEVs was 82.9% at pH 5.5 at 24 h, which was 1.55 times greater than that at pH 7.4.

To measure the in vivo biodistribution and biosafety of the AEVs, BALB/c mice were generated by the administration of DiD-labeled AEVs or saline (Fig. 1H). At 1, 4, 12, and 24 h following intravenous injection, DiD-AEVs showed no specific aggregation site in the body (Fig. 1I). After the mice were euthanized, their heart, liver, spleen, lung, kidney, and brain tissues were harvested for ex vivo imaging, and no signal was detected in these tissues (Fig. 1J). Next, the mice were subjected to blood biochemistry analysis as well as histological examination. No statistically significant differences were detected in liver function or kidney function (Fig. 1K). Finally, the heart, liver, spleen, lung, kidney, and brain tissues were collected for hematoxylin & eosin (H&E) staining. No apparent organ or tissue damage was observed in the AEVs and control groups (Fig. 1L). To comprehensively assess the biosafety of AEVs, an acute toxicity study was conducted in ICR mice administered escalating doses (50–800 mg/kg). Throughout the 14-day observation period, no mortality or significant body weight fluctuations were observed across all treatment groups (Supplementary Fig. S7A), suggesting excellent systemic tolerance. Consistent with these findings, histopathological examination of major organs—including heart, liver, spleen, lung, and kidney—revealed intact tissue architecture in both AEV-treated and control groups, with no evidence of inflammation, necrosis, or other pathological alterations (Supplementary Fig. S7B). The absence of dose-dependent toxicity in these critical organs, even at the highest administered concentration (800 mg/kg), further confirms the favorable safety profile of AEVs observed in previous biodistribution studies. The above results indicated that AEVs with improved biosafety were successfully obtained from fresh *A. annua*.

### scRNA-seq analysis of AEVs-induced inhibition of the growth of transplanted BC in mice

**Fig. 2:**
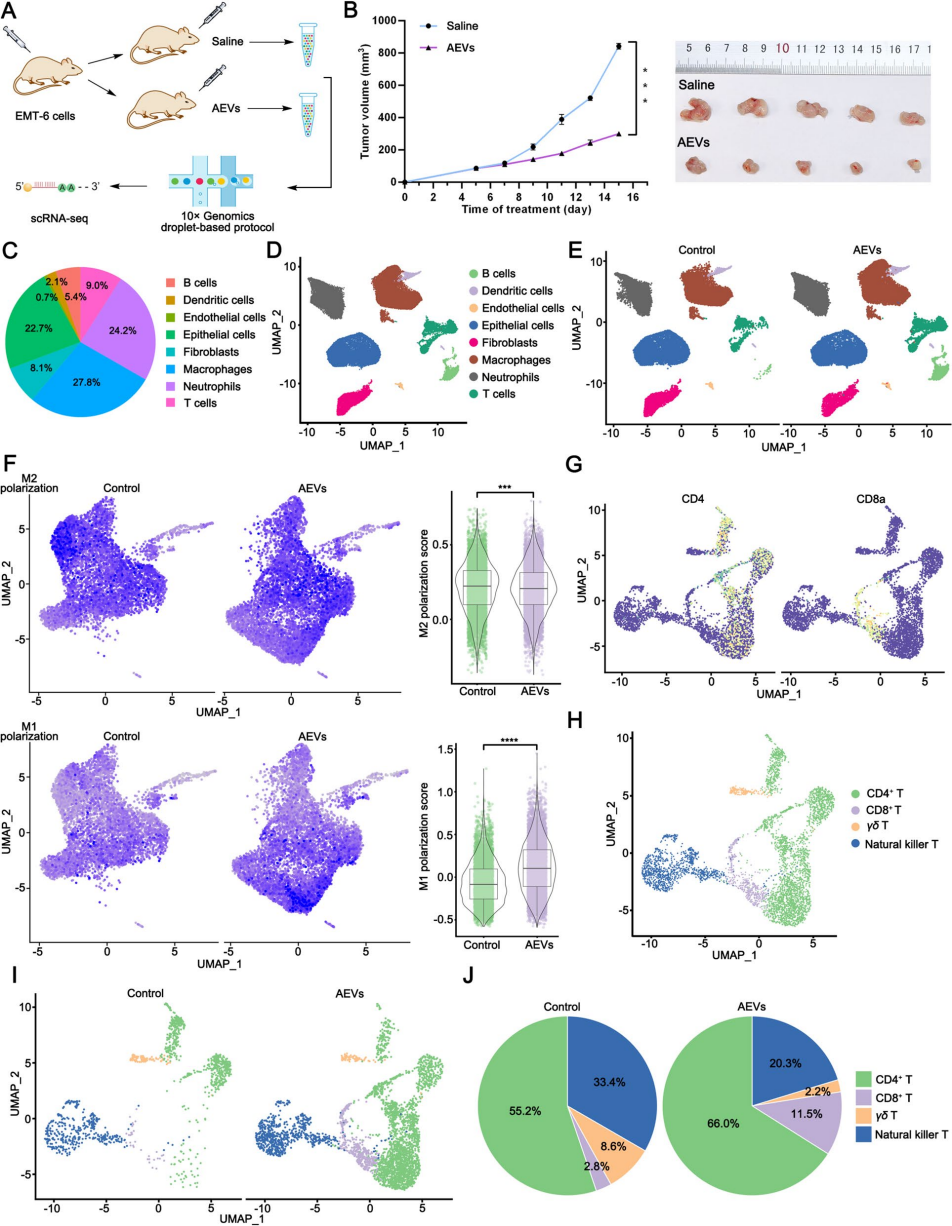
Single-cell RNA sequencing (scRNA-seq) analysis of AEVs inhibiting the growth of EMT-6 tumor. **A** Schematic diagram of scRNA-seq in EMT-6 tumor-bearing mice. **B** Tumor growth curves and photographs of tumor tissues after treatment of AEVs in EMT-6 tumor-bearing mice. Data were mean ± SD (n = 5). ^***^p < 0.001. **C**–**E** Subgroup clustering and proportion with or without treatment of AEVs. **F** The M1/M2-like polarization of macrophages after treatment of AEVs. ^***^p < 0.001. **G** Expression of sample marker genes. **H**–**J** T lymphocyte clustering and proportion with or without treatment of AEVs

To investigate whether AEVs exhibited antitumor effects, an EMT-6 cells-bearing mice model was established (Fig. 2A). After 15 days of treatment, the tumor growth of mice treated with AEVs was significantly inhibited compared with that of the saline-treated control mice (Fig. 2B). Next, to further reveal the specific mechanism by which AEVs inhibit tumor growth, we analyzed tumor growth using the scRNA-seq (10 × Genomics). After quality control and filtering (Supplementary Fig. S8), approximately 10000 cells of each group met the preprocessing threshold and were used for downstream analysis (Supplementary Fig. S9). The cell population in the TME was divided into eight major cell types based on their gene expression signatures (Supplementary Fig. S10), including B cells, dendritic cells, endothelial cells, epithelial cells, fibroblasts, macrophages, neutrophils, and T cells (Supplementary Fig. S10, Fig. 2C and D). We found that macrophages (27.8%), neutrophils (24.2%), and T cells (9.0%) mainly infiltrated tumor tissues. Compared with those in the control group, AEVs significantly increased the number of T cells and B cells and significantly decreased the number of fibroblasts (Fig. 2E, Supplementary Fig. S11). However, the macrophage population remained virtually unchanged. Gene Ontology (GO) analysis was used to perform enrichment analysis of the functions of the genes with altered expression (Supplementary Fig. S12). The altered genes were enriched for positive regulation of macrophage chemotaxis, the T-cell receptor signaling pathway, CD4 receptor binding, and CD8 receptor binding, which are related to the regulation of macrophages and T cells.

TAM infiltration in tumor tissues has been shown to be involved in tumor progression [10, 11]. Thus, macrophages were further divided into eight subclusters on the basis of gene expression (Supplementary Figs. S13 and S14,). The number of infiltrating M1-like macrophages in tumors significantly increased after AEVs treatment, whereas the number of M2-like macrophages significantly decreased (Fig. 2F). Next, T cells were divided into four subclusters on the basis of gene expression (Fig. 2G and H, Supplementary Figs. S15 and S16). CD4, Tnfrsf4, Trib2, St8sia1, and Cd5 were highly expressed in CD4^+^ T cells, whereas the levels of Cd8a, Cd8b1, Fam241a, Jaml, and Cd226 were greater in CD8^+^ T cells. After treatment with AEVs, the percentage of CD8^+^ T cells increased from 2.8% to 11.5% and the percentage of CD4^+^ T cells also increased from 55.2% to 66.0% (Fig. 2I and J). On the basis of these data, we speculated that the inhibition of tumor growth by AEVs was closely related to the regulation of macrophage polarization and the recruitment of T cells.

### AEVs alter the M2-like polarization of macrophages to exhibit antitumor effects

**Fig. 3:**
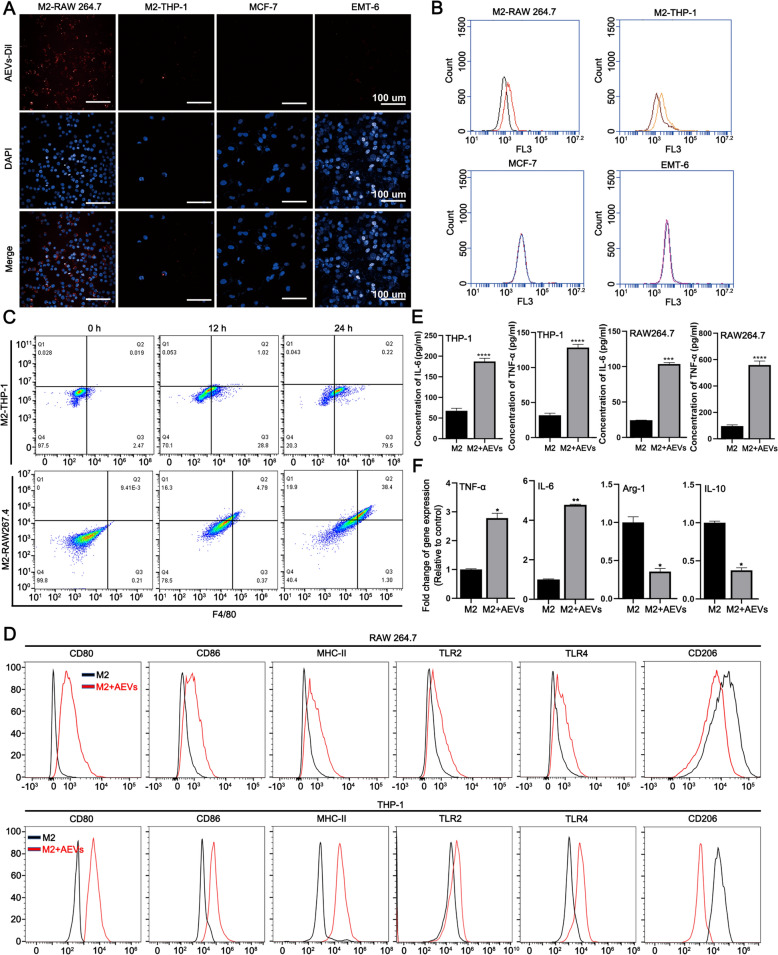
AEVs altering M2-like polarization of macrophages. **A** Fluorescence images of cells after treatment with Dil-AEVs. Scale bars = 100 μm. **B** Flow cytometry analysis of cells after treatment with Dil-AEVs. **C** Uptake efficiency of M2-like macrophages after treating Dil-AEVs by using flow cytometry. **D** Surface marker expression profile of M2-like macrophages treated with or without AEVs by using flow cytometry. **E** Levels of IL-6 and TNF-α in the supernatants of M2-like macrophages treated with or without AEVs by ELISA analysis. Data were mean ± SD (n = 3). ^***^p < 0.001 and ^****^p < 0.0001. **F** Relative mRNA levels of M1/M2-marker genes in M2-RAW264.7 cells after treatment with AEVs determined by qRT-PCR analysis. Data were mean ± SD (n = 3). ^*^p < 0.05 and ^**^p < 0.01

To further address the potential role of macrophages in the antitumor effect of AEVs, we first examined whether AEVs can be taken up by macrophages. THP-1 and RAW264.7 cells were incubated with IL-4 and IL-13 for 24 h to polarize the cells to an M2-like phenotype. M2-THP-1, M2-RAW264.7, MCF-7, and EMT-6 cells were incubated with AEVs labeled with Dil, a fluorescent red dye, for 12 h. Compared with that in tumor cells, red fluorescence (Dil-AEVs) was observed and preferentially localized in the cytoplasm of M2-THP-1 and M2-RAW264.7 cells (Fig. 3A). Flow cytometry showed the same results (Fig. 3B). The percentage of cells containing AEVs increased over time within 24 h among M2-THP-1 and M2-RAW264.7 cells (Fig. 3C, Supplementary Fig. S17). Thus, macrophages can take up AEVs in a time-dependent manner. Next, we determined whether AEVs can alter the M2-like polarization of macrophages. Flow cytometric analysis revealed that AEVs significantly increased the expression of CD80, CD86, MHC-II and TLR2/4, and reduced the level of CD206 in M2-like macrophages (Fig. 3D, Supplementary Fig. S18-19). The markers CD80, CD86, MHC-II, TLR2/4, and CD206 are polarization-related surface markers, that are characteristic of M1/M2 macrophages [26]. To further verify that AEVs can alter M2-like polarization, the production of cytokines by macrophages in the medium before and after AEVs treatment was quantified. ELISAs revealed that AEVs significantly increased the levels of M1 markers (IL-6 and TNF-α) (Fig. 3E). Transcriptional analysis corroborated these findings, with qRT-PCR demonstrating upregulated IL-6 and TNF-α mRNA expression concurrent with downregulation of characteristic M2 markers (Arg-1 and IL-10) (Fig. 3F). Functional characterization extended these observations, showing that AEV treatment enhanced macrophage phagocytic capacity by 8.9-fold compared to controls (Supplementary Fig. S20A). Parallel measurements of oxidative metabolism revealed a 1.7-fold increase in DCFH-DA-detected ROS generation, consistent with classical macrophage activation (Supplementary Fig. S20B). These data revealed that AEVs effectively induced M2-like polarization to M1-like polarization in macrophages in vitro.

**Fig. 4:**
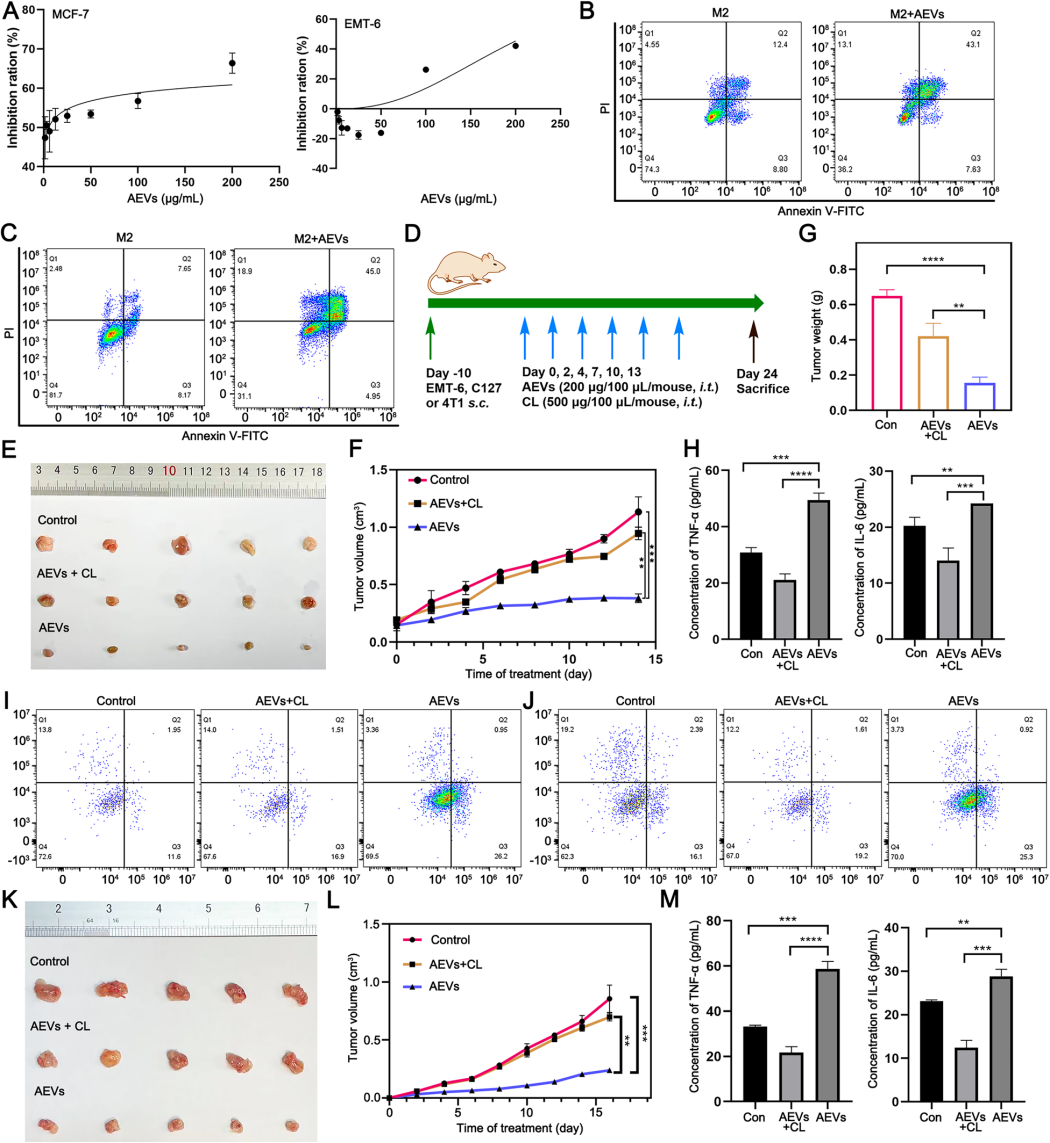
AEVs exhibiting anti-tumor in vitro and in vivo. **A** Inhibition ratio of cell culture medium supernatants to BC cells by MTT assay. The supernatants were collected after AEVs incubated M2-like macrophages. Data were the mean ± SD (n = 3). **B** Apoptotic rate of EMT-6 cells after treatment of medium supernatants determined by annexin V-FITC/PI staining. The supernatants were collected from M2-RAW264.7 cells or AEVs-treated M2-RAW264.7 cells. **C** Apoptotic rate of MCF-7 cells after treatment of medium supernatants determined by annexin V-FITC/PI staining. The supernatants were collected from M2-THP-1 cells or AEVs-treated M2-THP-1 cells. **D** Time schedule for molding and drug treatment in BC tumor-bearing mice. **E** Photographs of tumor tissues from EMT-6 tumor-bearing mice excised on day 14. **F** Tumor growth curves of EMT-6 Tumor-bearing mice over 14 days. Data were mean ± SD (n = 5). ^***^p < 0.001. **G** Tumor weight after administration on day 14 in EMT-6 tumor-bearing mice. Data were mean ± SD (n = 5). ^****^p < 0.0001. **H** Levels of IL-6 and TNF-α in the tumor tissues of EMT-6 tumor-bearing mice by ELISA analysis. Data were mean ± SD (n = 5). ^**^p < 0.01 and ^***^p < 0.001. **I**, **J** Representative flow cytometry analysis of M1 (CD86^+^) and M2 (CD206^+^) cell populations in immune microenvironment (CD45^+^) from EMT-6 tumor-bearing mice (**I**) or C127 tumor-bearing mice (**J**). **K** Photographs of tumor tissues from C127 tumor-bearing mice excised on day 16. **L** Tumor growth curves of C127 tumor-bearing mice over 16 days. Data were mean ± SD (n = 5). ^***^p < 0.001. **M** Levels of IL-6 and TNF-α in the tumor tissues of C127 tumor-bearing mice by ELISA analysis. Data were mean ± SD (n = 5). ^**^p < 0.01 and ^***^p < 0.001

Next, the proliferation inhibition rate and induced apoptosis rate of tumor cells were calculated to determine whether M2-like macrophages could kill tumor cells after being polarized to the M1-like phenotype. M2-like macrophages were incubated with or without different concentrations of AEVs for 48 h. Then, the supernatant medium was obtained and further incubated with tumor cells (MCF-7 or EMT-6). A 3-(4,5-dimethylthiazol-2-yl)−2,5-diphenyltetrazolium bromide (MTT) assay revealed that the supernatant exhibited dose-dependent cytotoxicity toward MCF-7 and EMT-6 cells (Fig. 4A). However, AEVs exhibited mostly nontoxic properties toward tumor cells with cell viability greater than 90% (Supplementary Fig. S21). Apoptosis induction was also assessed using an Annexin V-FITC/PI apoptosis assay detection kit. Compared with that of the M2 group (12.4%), the supernatant medium of the M2 + AEVs group induced a greater percentage of apoptotic MCF-7 cells (43.1%) (Fig. 4B and Supplementary Fig. S22). Similar results were observed for EMT-6 cells (Fig. 4C and Supplementary Fig. S22). Then, in vivo assays were carried out to study the antitumor effects of AEVs treatment through altering the M2-like polarization of macrophages. EMT-6 or C127 cell-bearing mice were constructed and randomly divided into three groups, which were treated with saline (control), AEVs, or AEVs + clodronate liposome (CL), respectively (Fig. 4D). CL was commonly used to clear macrophages [27], and our flow cytometry analysis confirmed this effect, showing CL treatment reduced TAMs from 42.4% to 16% without affecting neutrophil or dendritic cell populations (Supplementary Fig. S23). Compared with the control group, the AEVs group presented remarkably greater inhibition of tumor growth, whereas the AEVs + CL group presented unsatisfactory tumor inhibition during 14 days of treatment in EMT-6 cell-bearing mice (Fig. 4E and F). The lowest tumor weight was obtained in the AEV group, and the tumor inhibition rates in the AEVs and AEVs + CL groups were 66.3% and 25.6%, respectively, at 14th day of treatment (Fig. 4G, Supplementary Fig. S24). Furthermore, IL-6 and TNF-α expression significantly increased after treatment with AEVs, whereas their levels decreased in the AEVs + CL group (Fig. 4H). To analyze the macrophage populations of tumor tissues, multicolour flow cytometry with immunofluorescence was used. Tumor-infiltrating leukocytes were first identified by using anti-CD45 antibodies. Then, the macrophages were sorted using antibodies (F4/80^+^ and CD11b^+^). Compared with that in the control group, the percentage of M1 cells (CD86^+^) significantly increased from 11.6% to 26.2% in the AEVs group, whereas the percentage of M2-like macrophages (CD206^+^) decreased from 13.8% to 3.36% (Fig. 4I and Supplementary Fig. S25). H&E analysis, TUNEL assay, and Ki67 immunohistochemical analysis of tumor tissues further confirmed that the AEVs group exhibited better antitumor effects than did the AEVs + CL group (Supplementary Fig. S24). Moreover, in an in vivo C127 cell-bearing mice model, treatment with the control, AEVs, and AEVs + CL, respectively, produced similar results (Fig. 4J–M, Supplementary Fig. S24–25). The above data revealed that AEVs could shift the polarization of macrophages from the M2-like phenotype to the M1-like phenotype. The antitumor effect of AEVs was significantly reduced when macrophages were eliminated.

### AEVs activate NF-κB and inhibit the PPARγ signaling pathway to regulate macrophage polarization

**Fig. 5:**
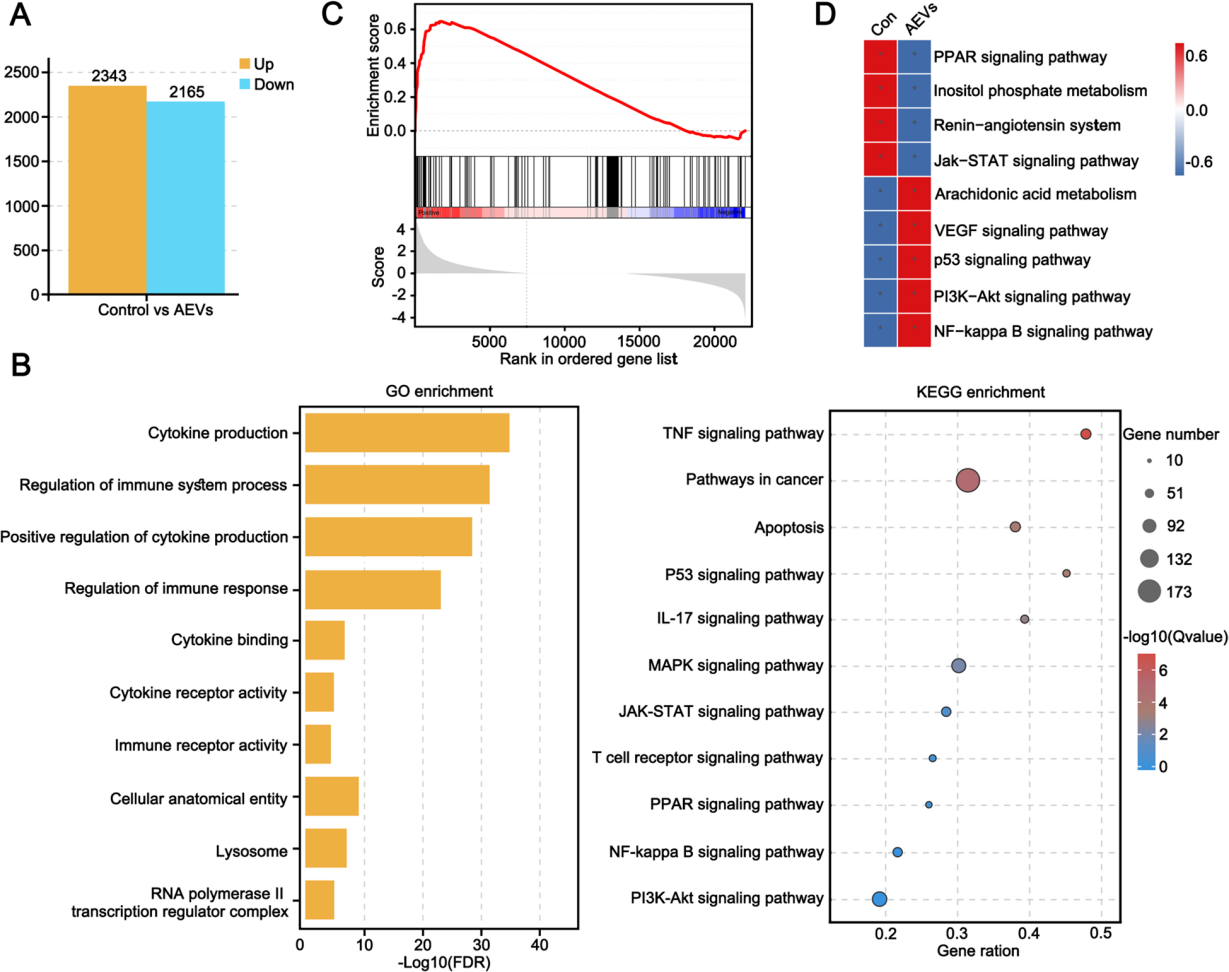
**A** Differential genes statistics of the mRNA-seq analysis of M2-RAW264.7 cells and AEVs-treated M2-RAW264.7 cells. **B** GO terms of the differentially expressed genes and KEGG pathway enrichment in AEVs-treated M2-RAW264.7 cells compared with M2-RAW264.7 cells. **C** Gene set enrichment analysis (GSEA) of NF-κB signaling pathway in mRNA-seq assay. **D** Gene set variation analysis (GSVA) of significant hallmark pathways in scRNA-seq assay

To further explore the mechanism by which AEVs alter the M2-like to M1-like polarization of macrophages, mRNA-seq technology was used to search for factors or genes that mediate macrophage polarization. After quality control and filtering (Supplementary Fig. S26), 2,343 genes were significantly upregulated and 2,165 genes were markedly downregulated in M2-RAW264.7 macrophages after treatment with AEVs (Fig. 5A, Supplementary Fig. S27). GO analysis revealed that genes with expression changes enriched in the immune response and regulation of the immune system (Fig. 5B). Kyoto Encyclopedia of Genes and Genomes (KEGG) enrichment analysis was used to analyze the distribution of genes or proteins in biological metabolic pathways or functional classifications. Through KEGG analysis of the mRNA-seq (Fig. 5B) and scRNA-seq data (Supplementary Fig. S12), we found that the PPARγ signaling pathway was notably inhibited and that the NF-κB signaling pathway was activated after treatment with AEVs (Fig. 5C and D).

**Fig. 6:**
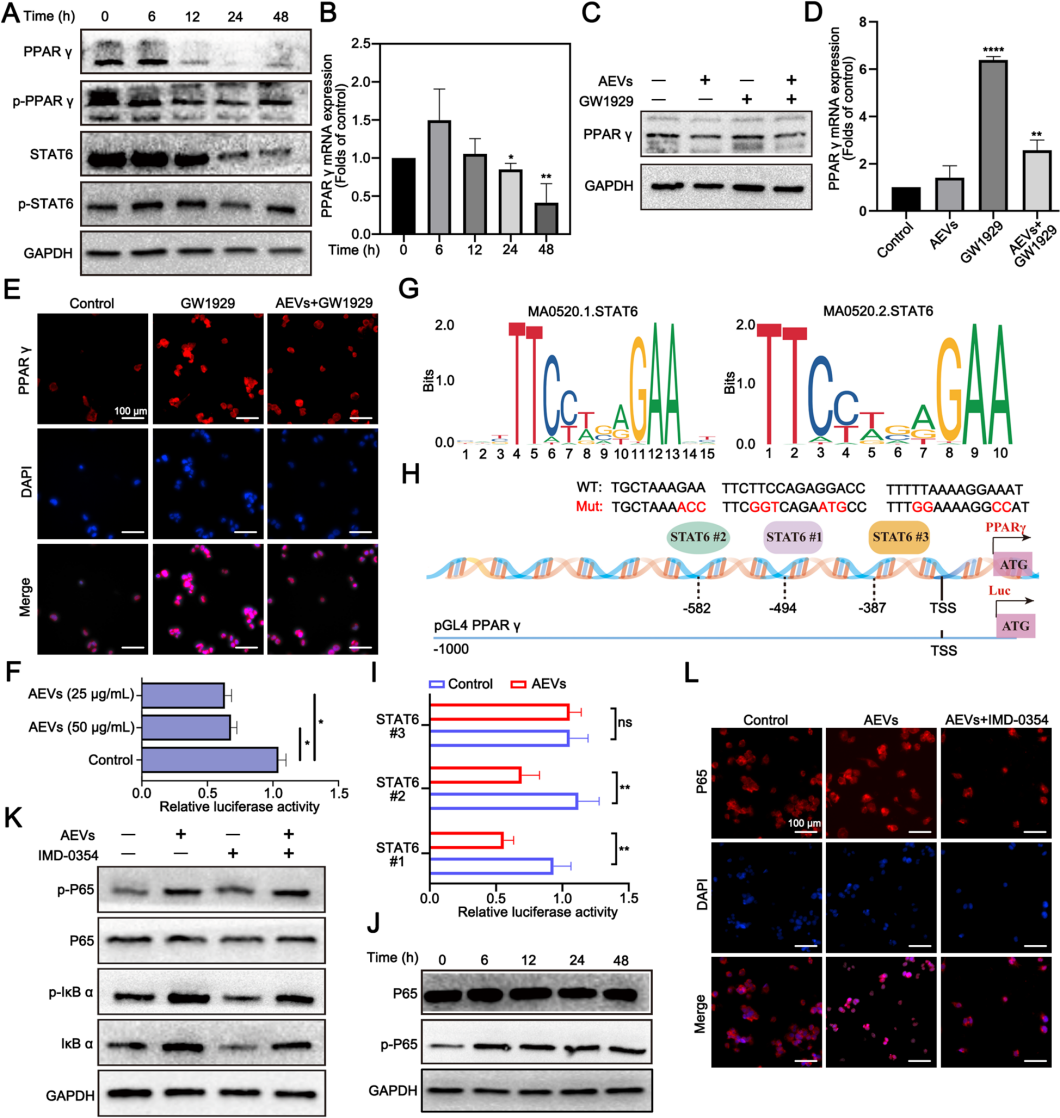
AEVs activating NF-κB and inhibiting PPARγ signaling pathway in M2-like macrophages. **A** Expression of PPARγ, p-PPARγ, p-STAT6 and STAT6 in M2-RAW264.7 cells after treatment with AEVs by Western blot analysis. **B** Relative mRNA levels of PPARγ in M2-RAW264.7 cells after treatment of AEVs by qRT-PCR analysis. Data were mean ± SD (n = 3). ^*^p < 0.05 and ^**^p < 0.01, compared to control. **C** Levels of PPARγ in M2-RAW264.7 cells after treatment by Western blot analysis. **D** Relative mRNA levels of PPARγ in M2-RAW264.7 cells after treatment by qRT-PCR analysis. Data were mean ± SD (n = 3). ^**^p < 0.01 and ^****^p < 0.0001, compared to control. **E** Fluorescence images of M2-RAW264.7 cells after treatment. Cells were stained with anti-PPARγ (red) and DAPI (blue), respectively. Scale bar = 100 μm. **F** Promoter activity of *PPARγ* gene after treatment of AEVs by luciferase reporter genes assay. Data were mean ± SD (n = 3), ^*^p < 0.05. **G** Predicted binding sites of STAT6 to PPARγ promoter by using JASPAR database (http://jaspar.genereg.net/). **H** Constructed mutations in STAT6 binding sites for luciferase reporter genes assay and schematic representation of the mutation of STAT6-binding sites on PPARγ promoter. **I** Transcriptional activity of STAT6 by luciferase reporter genes assay. Data were mean ± SD (n = 3). ^**^p < 0.01. **J** Expression of P65 and p-P65 in M2-RAW264.7 cells after treatment with AEVs by Western blot analysis. **K** Expression of P65, p-P65, IκBα, and p-IκBα in M2-RAW264.7 cells after treatment by Western blot analysis. **L** Fluorescence images of M2-RAW264.7 cells after treatment. Cells were stained with anti-P65 (red) and DAPI (blue), respectively. Scale bar = 100 μm

Next, to further confirm the inhibition of the PPARγ signaling pathway after treatment with AEVs, western blotting revealed that AEVs administration significantly decreased the levels of PPARγ and p-PPARγ (Fig. 6A, Supplementary Fig. S28A). qRT-PCR revealed that the mRNA level of PPARγ in M2-RAW264.7 cells also decreased dramatically (Fig. 6B). GW1929 is a potent PPARγ agonist [28]. After treatment with GW1929, the PPARγ level was 1.74 times greater than that in the control group. However, the level in the AEVs + GW1929 group was 0.56 times lower than that in the GW1929 group (Fig. 6C, Supplementary Fig. S28C). The PPARγ mRNA level results were similar to the above phenomena. Compared with that in the GW1929 group, the mRNA level of PPARγ in the AEVs + GW1929 group was dramatically lower (Fig. 6D). Next, immunofluorescence revealed that AEVs reduced the nuclear translocation of PPARγ. The fluorescence intensity of PPARγ in the AEVs + GW1929 group was approximately 63.8% of that in the GW1929 group (Fig. 6E, Supplementary Fig. S28E). Thus, these results indicated that AEVs inhibited PPARγ expression and thus affected its function. However, how AEVs regulate PPARγ required further study. We constructed a luciferase reporter gene plasmid containing an 800 bp PPARγ promoter region to test whether AEVs affect the binding of transcription factors to the PPARγ transcription region. Double luciferase reporter gene assay revealed that AEVs treatment significantly decreased the transcriptional activity of the PPARγ promoter (Fig. 6F). Signal transducer and activator of transcription 6 (STAT6) is an important transcription factor, that plays a crucial role in modulating the PPARγ signaling pathway [29]. Western blotting revealed that AEVs also decreased STAT6 and p-STAT6 expression in a time-dependent manner (Fig. 6A, Supplementary Fig. S28B). As shown in Fig. 6G, we predicted the binding sites of STAT6 to the PPARγ promoter using the JASPAR database (http://jaspar.genereg.net/). Furthermore, we constructed plasmids containing PPARγ promoter luciferase reporter genes with mutated STAT6 binding sites exist in the PPARγ promoter (Fig. 6H). After AEVs treatment, the relative luciferase activity of the STAT6-Mut1/2 binding sites decreased, whereas the activity of STAT6-Mut3 was almost unchanged (Fig. 6I). These results suggested that the STAT6-Mut3 binding site might play a key role in the AEVs-mediated transcriptional regulation of PPARγ.

Next, the ability of AEVs to induce NF-κB signaling pathway was further confirmed. Western blotting revealed that AEVs increased the phosphorylation level of P65 (p-P65/P65) in a time-dependent manner (Fig. 6J, Supplementary Fig. S28D). IMD-0354, as an inhibitor of IKKβ, is used to disrupt NF-κB signaling pathway [30]. After coincubating with IMD-0354, the expression of p-P65/P65 in the AEVs + IMD-0354 group was 1.87 times greater than that of the IMD-0354 group. Similarly, the p-IκB/IκB ratio in the AEVs + IMD-0354 group was 1.51 times greater than that in the IMD-0354 group (Fig. 6K, Supplementary Fig. S28F). Immunofluorescence revealed that AEVs increased the nuclear localization of P65 (Fig. 6L). The fluorescence intensity of P65 in AEVs + IMD-0354 group was approximately 56.2% of that in the AEVs group (Supplementary Fig. S28G). Moreover, qRT-PCR assay revealed that the mRNA level of P65 after treatment with AEVs also increased dramatically (Supplementary Fig. S28H). Taken together, these data suggest that AEVs can activate the NF-κB signaling pathway and simultaneously inhibit the PPARγ signaling pathway to regulate macrophage polarization.

### AEVs promote T cell infiltration by inducing the polarization of macrophages

**Fig. 7:**
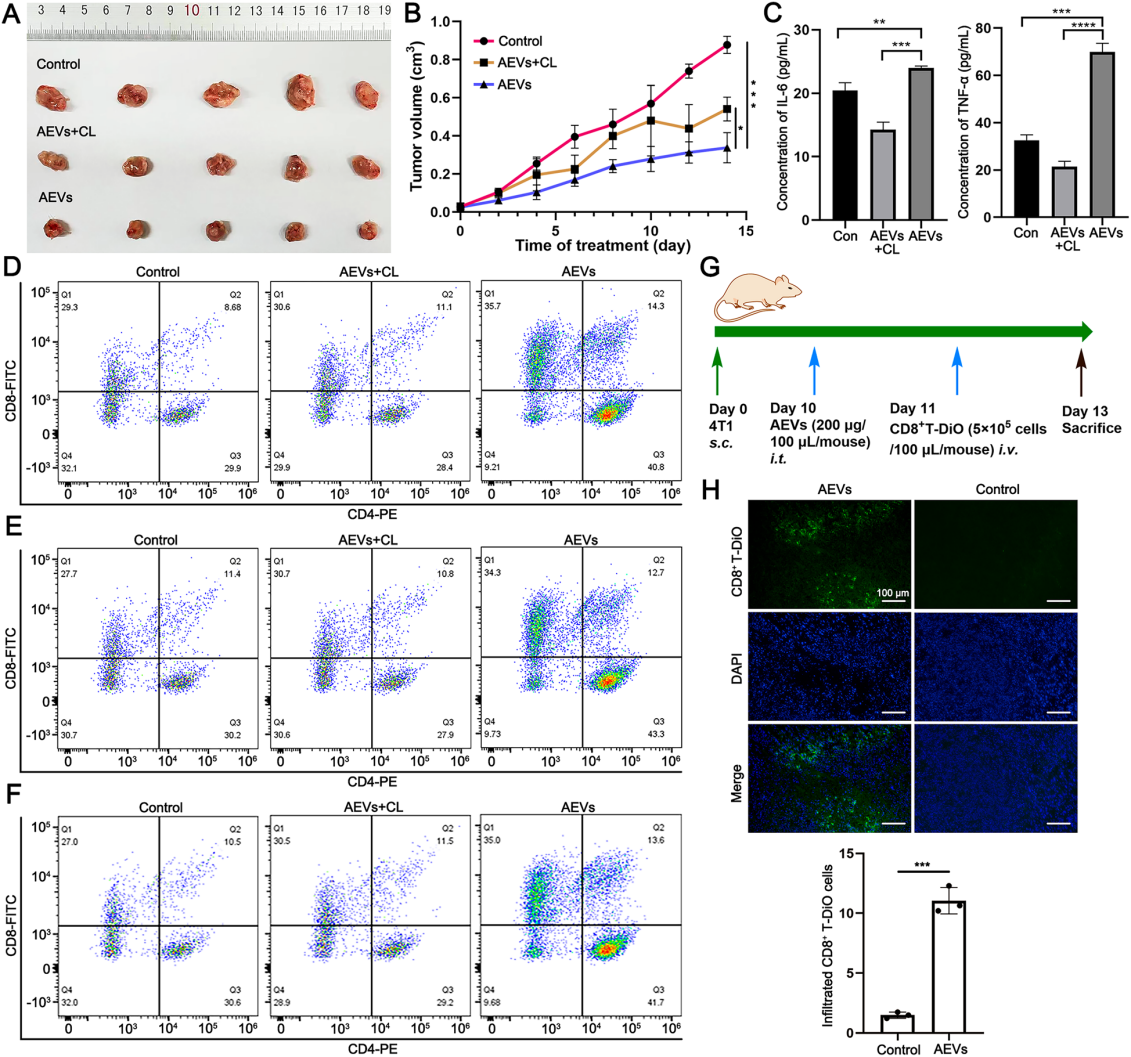
AEVs recruiting T lymphocytes infiltration. **A** Photographs of tumor tissues from 4T1 tumor-bearing mice excised on day 14. **B** Tumor growth curves of 4T1 tumor-bearing mice over 14 days. Data were mean ± SD (n = 5). ^*^p < 0.05 and ^***^p < 0.001. **C** Levels of IL-6 and TNF-α in the tumor tissues of 4T1 tumor-bearing mice by ELISA analysis. Data were mean ± SD (n = 5). ^**^p < 0.01 and ^***^p < 0.001. **D**–**F** Representative flow cytometry analysis of CD4^+^ T (CD4^+^) and CD8^+^ T (CD8a^+^) cell populations in immune microenvironment (CD45^+^) from 4T1 tumor-bearing mice (**D**), EMT-6 tumor-bearing mice (**E**) or C127 tumor-bearing mice (**F**). **G** Time schedule for chemotaxis assay in 4T1 tumor-bearing mice in vivo. **H** Fluorescence images and quantitative analysis of infiltrative DiO-labeled CD8^+^ T lymphocytes with treatment of AEVs in 4T1 tumor-bearing mice. Scale bar = 100 μm. Data were mean ± SD (n = 3). ^***^p < 0.001

As previously confirmed, AEVs promote the polarization of M2-like macrophages into the tumoricidal M1 type; however, how AEVs-treated macrophages enhance T cell infiltration needed further study. We selected a cold tumor cell line (4T1 cell) to construct a 4T1 cell-bearing mice model. Three groups were randomly allocated and intratumorally injected with saline, AEVs + CL, or AEVs. As expected, compared with the other groups, the AEVs significantly suppressed tumor growth during 14 days of treatment (Fig. 7A–C, Supplementary Fig. S29). AEVs can alter the polarization of macrophages from the M2-like to the M1-like phenotype to restrain the growth of tumors in vivo, and this therapeutic effect is similar to that in EMT-6/C127 cell-bearing mice. More importantly, we used multicolor flow cytometry to analyze the infiltrating T cell groups in tumor tissues. CD3-positive cells (T cells) were first sorted out from CD45-positive cells (leukocytes). As shown in Fig. 7D and Supplementary Fig. S30, the ratio of CD8^+^ T cell counts (CD8a^+^/CD3^+^) was significantly increased from 29.3% to 35.7% in the AEVs group compared with that in the control group. Moreover, the ratio of CD4^+^ T cell counts (CD4^+^/CD3^+^) was also significantly increased from 29.9% to 40.8% in AEVs group than in control group. Similar results were also observed in EMT-6/C127 cell-bearing mice (Fig. 7E, F and Supplementary Fig. S30). To further clarify whether immune T cells were recruited after AEVs-treated macrophages, DiO-stained CD8^+^ T cells were intravenously injected into 4T1 tumor-bearing mice (Fig. 7G). Immunofluorescence staining data revealed that AEVs treatment recruited more CD8^+^ T cells in the tumor tissues than the control group (Fig. 7H).

**Fig. 8:**
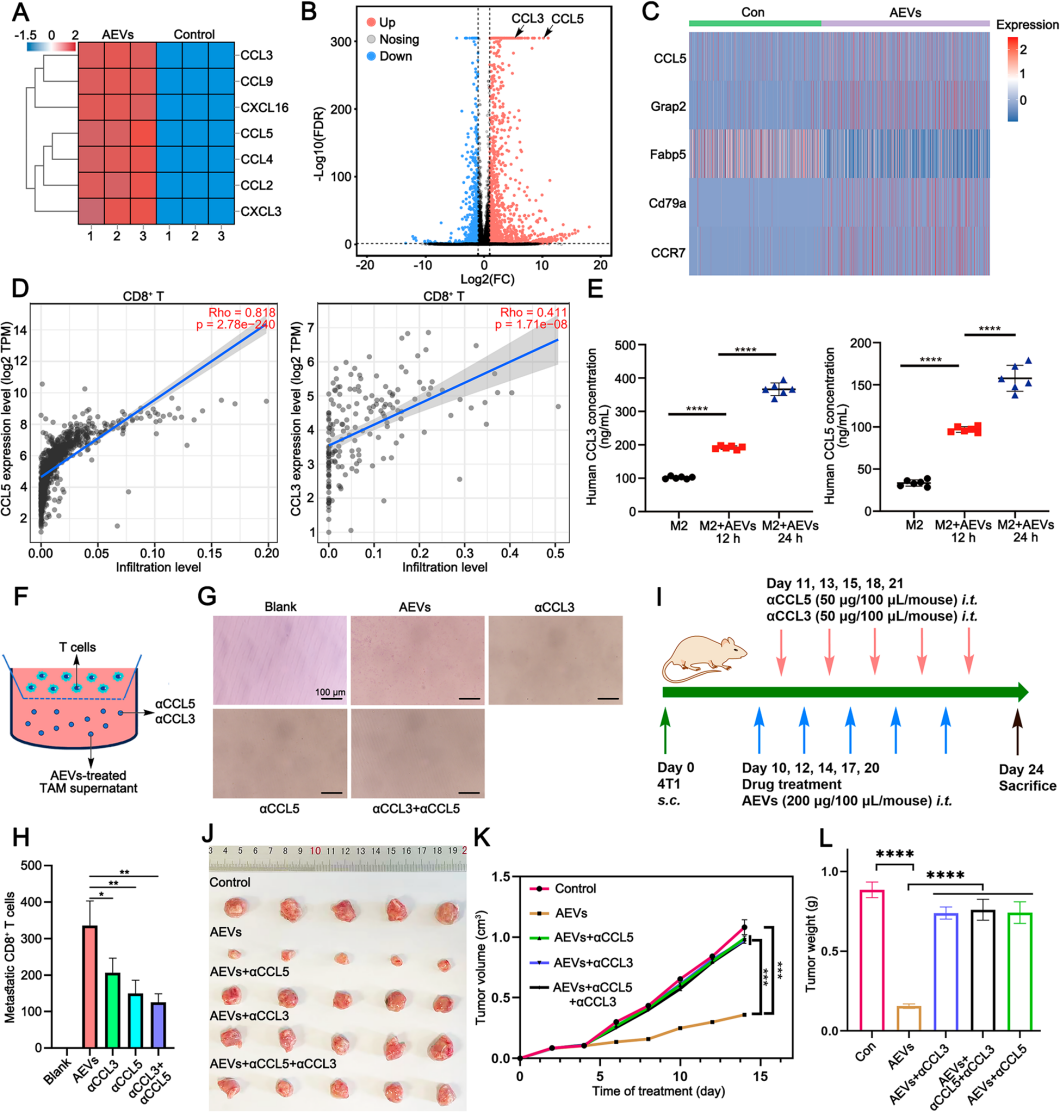
AEVs prompting macrophages to secrete chemokines. **A**, **B** Heat map (**A**) of representative gene expression and volcano plot (**B**) of mRNA-seq analysis of M2-RAW264.7 cells with or without treatment of AEVs. **C** Heat map of selected genes’ levels in scRNA-seq analysis. **D** Relationship between CCL5/CCL3 and CD8^+^ T‑cell infiltration level in BC analyzed by TIMER2.0 (http://timer.cistrome.org/). **E** CCL5 and CCL3 concentration in the culture media of M2-THP-1 cells after treatment of AEVs by ELISA analysis. Data were mean ± SD (n = 6). ^****^p < 0.0001. **F**–**H** Schematic diagram, photographs and quantification of chemotactic assay in vitro. CD8^+^ T cells migrated toward the supernatants of AEVs-treated M2-THP-1 cells in the presence of αCCL5 and αCCL3 neutralization by flow cytometry. Data were mean ± SD (n = 3). ^*^p < 0.05 and ^**^p < 0.01. **I** Time schedule for molding and drug treatment in 4T1 tumor-bearing mice by neutralization assay in vivo. **J** Photographs of tumor tissues from 4T1 tumor-bearing mice of neutralization assay excised on day 14. **K** tumor growth curves of 4T1 tumor-bearing mice over 14 days. Data were mean ± SD (n = 5). ^***^p < 0.001. **L** Tumor weight of 4T1 tumor-bearing mice after treatment at day14. Data were mean ± SD (n = 5). ^***^p < 0.001

The above research data revealed that AEVs optimized the immune microenvironment by increasing T cells infiltration. Thus, we further explored how AEVs-treated macrophages enhances T cell infiltration in the immune microenvironment. The changes of chemokines after AEVs stimulation were investigated. Heat map of representative gene expression and volcano plot of mRNA-seq analysis of M2-RAW264.7 cells revealed that the expression of CCL5 and CCL3 were increased after treatment with AEVs (Fig. 8A and B). This conclusion was further confirmed by the scRNA-seq analysis with or without AEVs in vivo (Fig. 8C). The correlations between CCL5/CCL3 and immune cells (CD8^+^ T cells) were analyzed by TIMER2.0 (https://cistrome.shinyapps.io/timer/). The results revealed that CCL5 and CCL3 were positively correlated with tumor infiltrating CD8^+^ T cells in BC (Fig. 8D). Moreover, higher expression of CCL5 and CCL3 in BC patients was associated with longer survival according to the GEPIA database (http://gepia.cancer-pku.cn/) (Supplementary Fig. S31). The levels of CCL5 and CCL3 in the culture media of M2-RAW264.7 cells with or without AEVs in vitro were further analyzed. ELISA assays confirmed that AEVs dramatically increased secretion of CCL5 and CCL3 in time-dependent manner (Fig. 8E). To further confirm the importance of CCL5 and CCL3 in recruiting infiltrating CD8^+^ T cells, chemotactic assays were performed using neutralizing antibodies (αCCL5 and αCCL3) to inhibit CCL5 and CCL3 (Fig. 8F and Supplementary Fig. S32). The flow cytometry results revealed that the numbers of metastatic CD8^+^ T cells in the αCCL5, αCCL3, and αCCL5 + αCCL3 groups were approximately 0.45, 0.62, and 0.37 times lower than that in the AEVs group, respectively (Fig. 8G and H). Finally, we constructed a 4T1 tumor-bearing mice model to verify the effects of CCL5 and CCL3 in vivo (Fig. 8I). Compared with the AEVs group, the AEVs + αCCL5, AEVs + αCCL3, and AEVs + αCCL5 + αCCL3 groups did not effective inhibition of tumor growth (Fig. 8J and K). After treatment for 14 days, the mice were euthanized, and the tumors were collected. The tumor weights of the AEVs group were 0.20-, 0.21- and 0.20-fold lower than those of the AEVs + αCCL5, AEVs + αCCL3, and AEVs + αCCL5 + αCCL3 groups, respectively (Fig. 8L). These findings suggests that AEVs induce macrophages to secrete CCL5 and CCL3, which in turn recruite more CD8^+^ T cells to infiltrate the tumor immune microenvironment.

## Discussion

Breast cancer (BC) remains the most prevalent malignancy among women worldwide and a leading cause of cancer-related mortality [31]. Emerging evidence underscores the critical role of the immune system in modulating BC progression through immunoediting, wherein tumor cells evade immune surveillance by suppressing immune responses or fostering a pro-tumorigenic inflammatory milieu [5, 32]. Targeting the immunosuppressive tumor microenvironment has thus emerged as a promising therapeutic strategy.

*A. annua*, as a well-known Chinese herb, is recorded in “Principal Prescriptions for Emergency (Zhou Hou Bei Ji Fang),” which was written by Ge Hong in 340 AD during China's Eastern Jin Dynasty. The extraction of a handful of fresh *A. annua* with approximately 400 mL of water provides the extracted juice, and the fresh juice helps to treat malaria. Inspired by this description that Tu Youyou, a senior Chinese pharmacologist, isolated artemisinin from *A. annua*, and won the 2015 Nobel Prize in Physiology or Medicine. Thus, fresh use of *A. annua* has been recorded since ancient times. Currently, *A. annua* is officially recognized as a medicinal plant and was listed in the 2020 Chinese Pharmacopeia. Recently, extracts of *A. annua* were reported to exert antitumor effects via immunoregulation, inducing cell growth cycle arrest, promoting apoptosis, and inhibiting the angiogenesis and tissue invasion of tumors [19–21]. We successfully isolated AEVs from the extracted juice of *A. annua*. Similar to other natural plant-derived EVs, AEVs have a stable nanovesicle shape and mainly contain lipids, amino acids and small molecules. Notably, the main active ingredient, artemisinin, is found in AEVs, whereas the extracted juice of *A. annua* without AEVs contains no artemisinin. Thus, AEVs provide a reference method for highly efficient enrichment of artemisinin. Additionally, we further demonstrated that AEVs could load exogenous artemisinin and that artemisinin-loaded AEVs could release more artemisinin in acidic solutions (pH 5.5) than in neutral solutions (pH 7.4) in vitro. AEVs may be used as nanocarriers for the targeted delivery of specific drugs for the treatment of certain diseases [33].

**Fig. 9:**
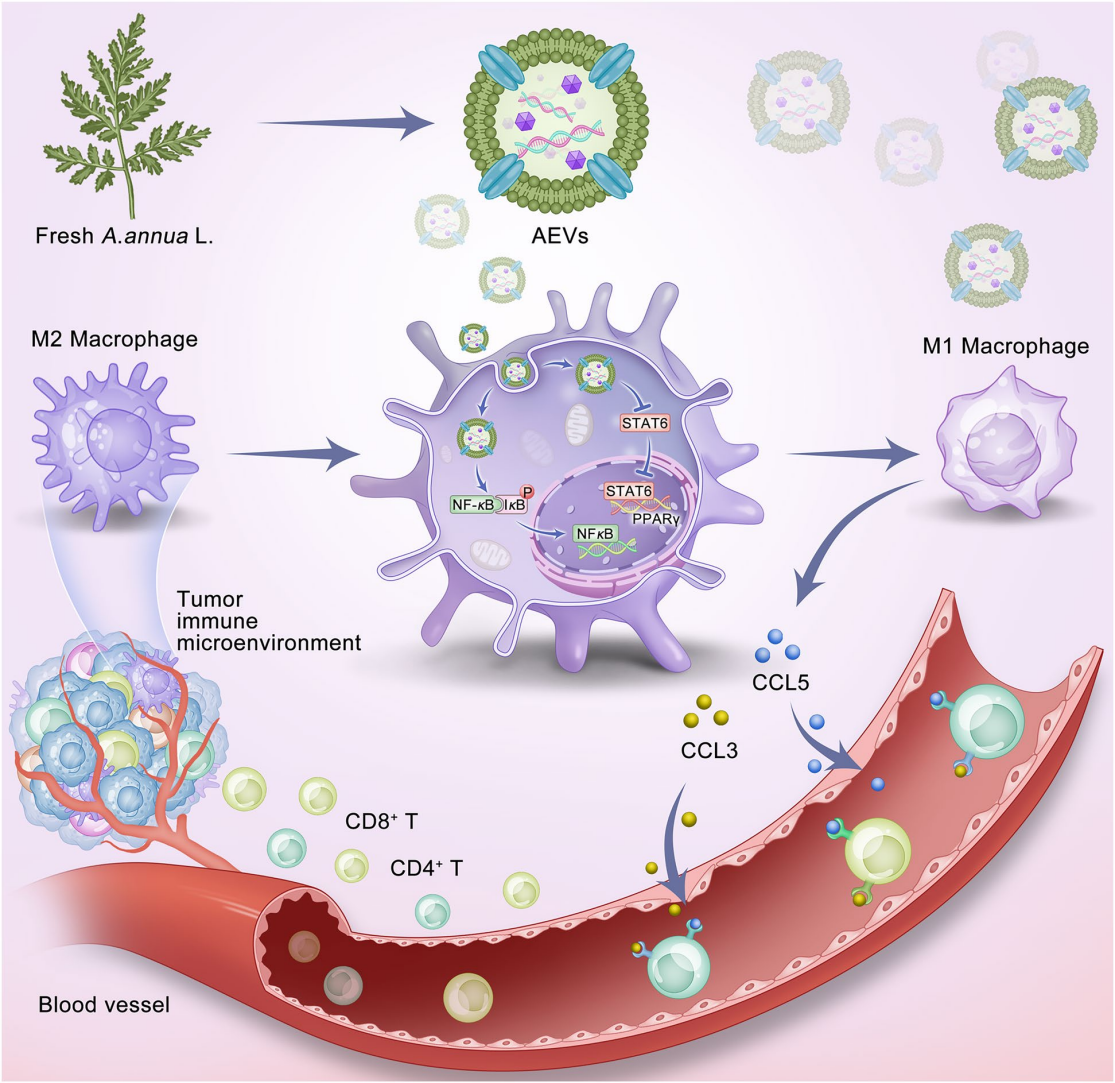
Schematic illustration of AEVs reprogramming breast tumor immune microenvironment via altering macrophage polarization and synergizing recruitment of T lymphocytes

AEVs can activate the NF-κB signaling pathway to polarize M2-like macrophages into M1-like macrophages. NF-κB is a critical transcription factor that facilitates the transcription of genes encoding proinflammatory cytokines to polarize macrophages and recruit immune cells to the immune microenvironment [34]. TAMs can produce different interleukins and factors, such as IL-6, IL-12, and TNF-α, through NF-κB-mediated signaling to induce the apoptosis of tumor cells [35]. In addition, AEVs can inhibit the PPAR signaling pathway in M2-like macrophages. The transcription factor PPARγ is instrumental in the process of macrophage polarization [36], and it controls the direction of macrophage polarization by promoting polarization to the M2-like phenotype and inhibiting polarization to the M1-like phenotype [37]. In macrophages, IL-4 can regulate lipid metabolism, and then upregulate the expression of PPARγ, driving macrophage polarization to the M2-like phenotype. Thus, our study revealed that AEVs can not only activate the NF-κB signaling pathway, but also cooperate to inhibit the PPARγ pathway to drive the polarization of macrophages from the protumor M2-like phenotype to the anti-tumor M1-like phenotype. Moreover, AEVs-induced macrophages produce chemokines CCL5 and CCL3 to promote the infiltration of T lymphocytes into tumors. There is increasing evidence that CD4^+^ and CD8^+^ T cells predominantly express CCR5, which is important for T lymphocyte infiltration in tumor beds [38]. CCL5, CCL3, and CCL4, which bind to CCR5, can induce the chemotaxis of activated/memory T cells but not that of naïve T cells [39]. These results indicate that AEVs can reprogram breast tumor immune microenvironment by altering macrophage polarization and synergizing recruitment of T lymphocytes (Fig. 9).

While these findings position AEVs as a promising immunomodulatory agent, several questions remain. The specific AEV components responsible for macrophage reprogramming require identification, and the contribution of artemisinin to these effects warrants further investigation. Future studies should also explore synergistic combinations with existing immunotherapies to enhance clinical applicability.

## Conclusion

In summary, AEVs were separated from fresh *A. annua* and identified as nanovesicles with diameters of approximately 137.2 nm. The AEVs included proteins, lipids, amino acids, and small molecules (including artemisinin). ScRNA-seq analysis revealed that AEVs inhibited tumor growth by altering macrophage polarization from the M2-like phenotype to the M1-like phenotype and synergizing with the recruitment of T lymphocytes in EMT-6 tumor-bearing mice. In M2-like macrophages, AEVs could activate the NF-κB signaling pathway while inhibiting the PPARγ signaling pathway to promote macrophage polarization. Concurrently, polarized M1-like macrophages secreted CCL5 and CCL3 to promote T lymphocyte infiltration. Thus, AEVs reversed the immunosuppressive effects by reshaping the BC immune microenvironment. Our findings open a new avenue for the medicinal use of fresh *A. annua* and provide a basis for BC immunotherapy.

## Supplementary Information

Supplementary Material 1

## Data Availability

No datasets were generated or analysed during the current study.

## Abbreviations

AEVs: *A. annua*-derived EVs
Arg-1: Arginase-1
BC: Breast cancer
CL: Clodronate liposome
EE: Encapsulation efficiency
EVs: Extracellular vesicles
GO: Gene ontology
H&E: Haematoxylin & eosin
IL-6: Interleukin-6
KEGG: Kyoto encyclopedia of genes and genomes
MS: Mass spectrometer
MTT: 3-(4,5-dimethylthiazol-2-yl)-2,5-diphenyltetrazolium bromide
PBS: Phosphate buffer saline
qRT-PCR: Quantitative real-time polymerase chain reaction
scRNA-seq: Single-cell RNA-seq
SD: Standard deviation
STAT6: Signal transducer and activator of transcription 6
TAMs: Tumor-associated macrophages
TME: Tumor microenvironment
TNF-α: Tumor necrosis factor α
