# Supplementary material for: Artemisia annua-derived extracellular vesicles reprogram breast tumor immune microenvironment via altering macrophage polarization and synergizing recruitment of T lymphocytes
Source: Chin Med. 2025 Oct 1;20:149. doi: 10.1186/s13020-025-01210-1 (PMC12487325; doi:10.1186/s13020-025-01210-1)
Supplement: Supplementary file 1 — Supplementary Material 1 [file 13020_2025_1210_MOESM1_ESM.docx]

**Supplementary material**

***Artemisia annua*-derived extracellular vesicles reprogram breast tumor immune microenvironment via altering macrophage polarization and synergizing recruitment of T lymphocytes**

Yun Wang^b,1^, Lin Meng^b,1^, Sicheng Su^b^, Yu Zhao^b^, Xiaoxian Hu^b^, Xiaoqing Xu^b^, Chao Han^b, *^, Jianguang Luo^b, *^, Zhongrui Li^a, *^

^a^ *Department of Rehabilitation, College of Acupuncture and Moxibustion and Massage Health Preservation and Rehabilitation, Nanjing University of Chinese Medicine, Nanjing 210023, PR China.*

^b^ *Basic Medical Research Innovation Center for Anti-Cancer Drugs, Jiangsu Key Laboratory of Bioactive Natural Product Research, School of Traditional Chinese Pharmacy, China Pharmaceutical University, Nanjing, 211198, China.*

^*^Corresponding authors.

E-mail addresses: Chao Han: hanchao@cpu.edu.cn, Jianguang Luo: luojg@cpu.edu.cn, Zhongrui Li: lizhongrui@njucm.edu.cn

^1^These authors made equal contributions to this work.

**Table S1.** Antibodies used for flow cytometry, immunofluorescence and Western blotting.

| **Antibody** | **Fluorophore** | **Company** | **Catalog No.** |
| --- | --- | --- | --- |
| Mouse CD206 | Brilliant Violet 421 | BioLegend | 141717 |
| Mouse CD80 | FITC | BioLegend | 104705 |
| Mouse CD86 | PE/Cy7 | BioLegend | 105013 |
| Mouse TLR2 | PE | BioLegend | 148603 |
| Mouse TLR4 | APC | BioLegend | 145403 |
| Mouse MHC-II | Brilliant Violet 605 | BioLegend | 107639 |
| Human CD206 | Brilliant Violet 421 | BioLegend | 321125 |
| Human CD80 | FITC | BioLegend | 305205 |
| Human CD86 | PE/Cy7 | BioLegend | 305421 |
| Human TLR2 | PE | BioLegend | 309707 |
| Human TLR4 | APC | BioLegend | 312815 |
| Human MHC-II | Brilliant Violet 605 | BioLegend | 307639 |
| Mouse CD45 | Brilliant Violet 421 | BioLegend | 103113 |
| Mouse CD11b | FITC | BioLegend | 101205 |
| Mouse CD4 | PE | BioLegend | 130310 |
| Mouse CD3 | APC | BioLegend | 100235 |
| Mouse CD8 | FITC | BioLegend | 100705 |
| Mouse F4/80 | PE/Cy7 | BioLegend | 123113 |
| Mouse CD86 | PE | BioLegend | 159203 |
| Mouse CD206 | APC | BioLegend | 162505 |
| PPARγ | - | Proteintech | 66826-1-Ig |
| p-PPARγ | - | Affinity | AF3284 |
| STAT6 | - | Proteintech | 51073-1-AP |
| p-STAT6 | - | Affinity | AF3301 |
| P65 | - | Proteintech | 10745-1-AP |
| p-P65 | - | Affinity | AF2006 |
| IκBα | - | Proteintech | 10268-1-AP |
| p-IκBα | - | Proteintech | 82349-1-RR |
| GAPDH | - | Proteintech | 60004-1-Ig |

**Table S2.** Primer sequences of mouse genes for qRT-PCR analysis.

| **Gene** | **Sequence (5’-3’)** |
| --- | --- |
| *GAPDH* | Forward TGAAGCAGGCATCTGAGGG |
|  | Reverse CGAAGGTGGAAGAGTGGGAG |
| *TNF-α* | Forward CCTCCCTCTCATCAGTTCTA |
|  | Reverse ACTTGGTGGTTTGCTACGAC |
| *IL-6* | Forward CCCCAATTTCCAATGCTCTCC |
|  | Reverse TTGGTCCTTAGCCACTCCTTC |
| *Arg-1* | Forward CAGAAGAATGGAAGAGTCAG |
|  | Reverse CAGATATGCAGGGAGTC |
| *IL-10* | Forward CTTACTGACTGGCATGAGGATCA |
|  | Reverse GCAGCTCTAGGAGCATGTGG |
| *PPARγ* | Forward GGAAGACCACTCGCATTCCTT |
|  | Reverse GTAATCAGCAACCATTGGGTCA |
| *NF-κB* | Forward ATGGCAGACGATGATCCCTAC |
|  | Reverse CGGAATCGAAATCCCCTCTGTT |

**Table S3.** Primer sequences for human genes used in double luciferase reporter gene assay.

| **Gene** | **Strand** | **Sequence (5'-3')** |
| --- | --- | --- |
| *PPARγ* | Forward | GTGCCAGAACATTTCTCTATCGATAGGTACCCCGGGTGAAGGGGTCTTGG |
|  | Reverse | GCTTTACCAACAGTACCGGAATGCCAAGCTTCCCTTCCTTACCTACCACTGACCTTATATAAAAGCCGCAGCTC |
| *STAT6*-Mut1 | Forward | CGAATGTTTGAAGACCGCAGTGAATTCGTGCCAGAACATTTCTCTATCGATAGGTACCTTTGT |
|  | Reverse | GATGCGTTTGAAGACTTACCATCCATGGGCTTTACCAACAGTACCGGAATGCCAAGCTTAC |
| *STAT6*-Mut2 | Forward | CGAATGTTTGAAGACCGCAGTGAATTCGTGCCAGAACATTTCTCTATCGATAGGTACCCTCC |
|  | Reverse | GATGCGTTTGAAGACTTACCATCCATGGGCTTTACCAACAGTACCGGAATGCCAAGCT |
| *STAT6*-Mut3 | Forward | CGAATGTTTGAAGACCGCAGTGAATTCGTGCCAGAACATTTCTCTATCGATAGGTACCCACA |
|  | Reverse | GATGCGTTTGAAGACTTACCATCCATGGGCTTTACCAACAGTACCGGAATGCCAAGCTTACTTAGATCGCAGATCTCGA |


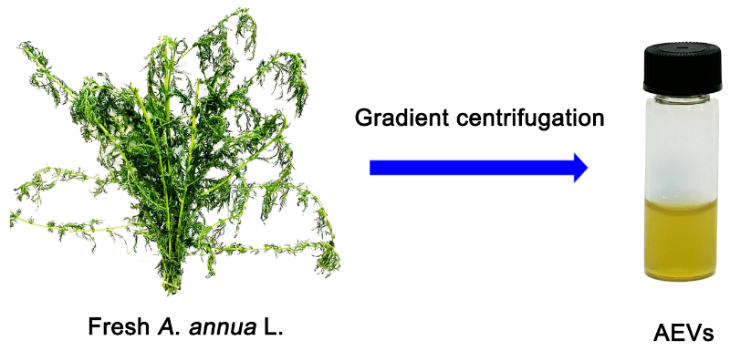


**Figure S1.** Separation of AEVs from fresh *A. annua* by gradient centrifugation.


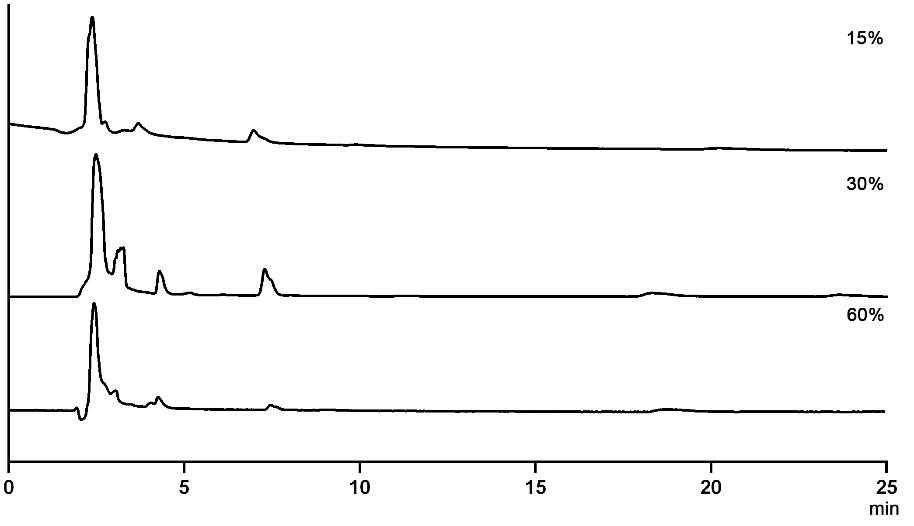


**Figure S2.** HPLC analysis of artemisinin distribution across sucrose gradient fractions (15%, 30%, and 60%).


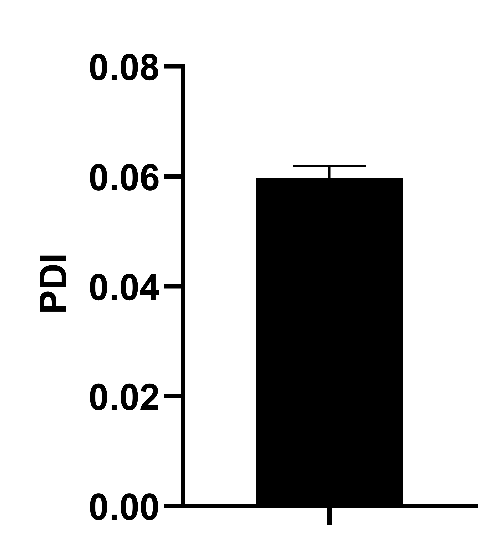


**Figure S3.** Polydispersity index (PDI) of AEVs. Data were mean ± SD (n = 3).


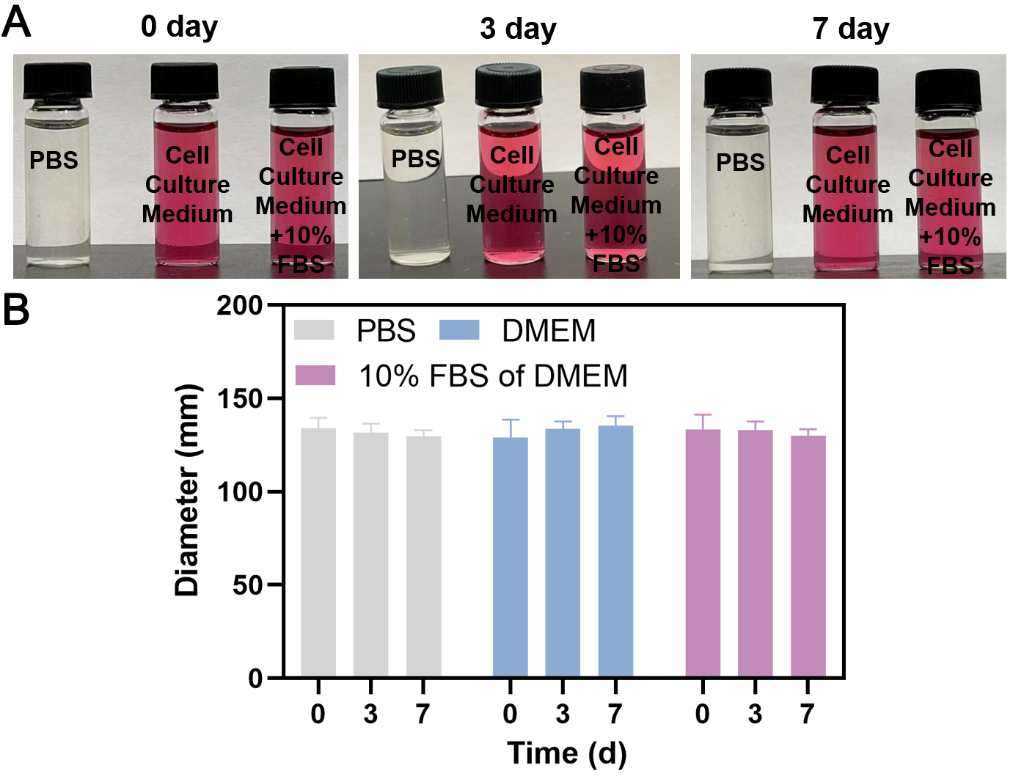


**Figure S4.** Images (A) and particle size (B) of AEVs in PBS, DMEM, and DMEM+10%FBS at day 0, 3 and 7. Data were mean ± SD (n = 3).


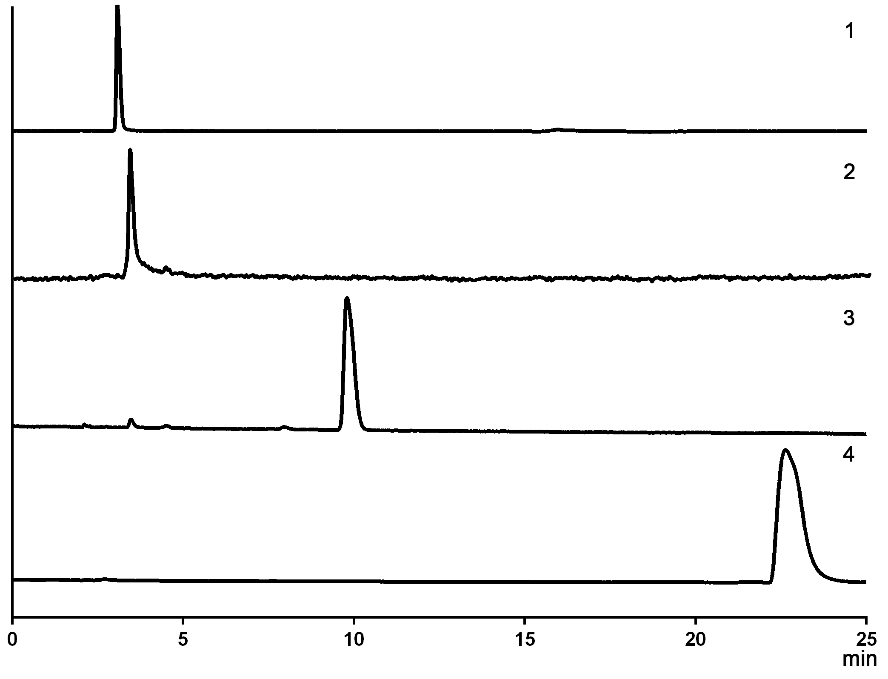


**Figure S5.** HPLC chromatograms of reference standards for bioactive compounds.

1-scopoletin, 2-apigenin, 3-chlorogenic acid, and 4-artemisinic acid.


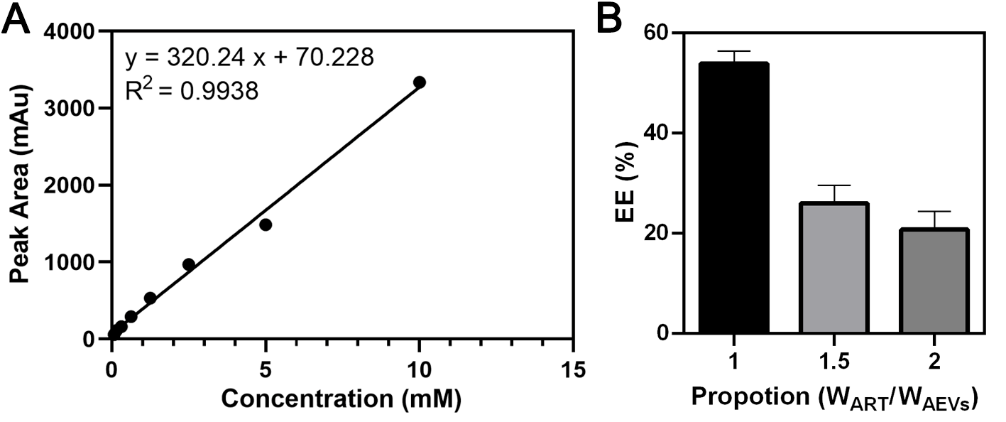


**Figure S6.** (A) Linear correlation between the peak area (210 nm) and the artemisinin (ART) concentration by using HPLC. (B) Encapsulation efficiency (EE) values of different weight ratios of ART/AEVs. Data were mean ± SD (n = 3).


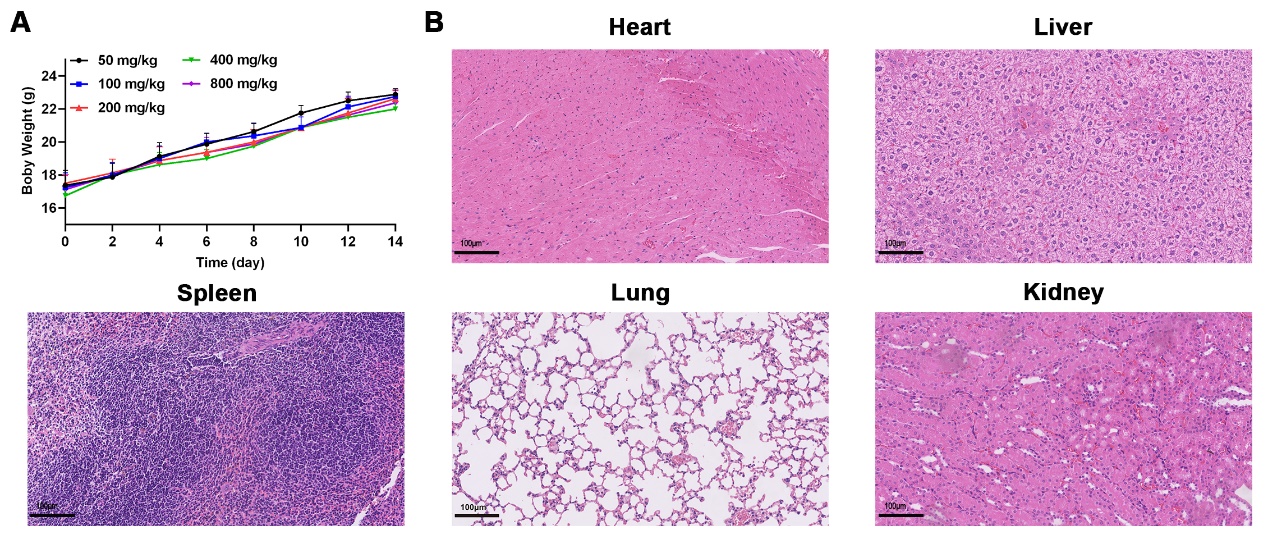


**Figure S7**. Evaluation of acute toxicity following AEVs administration. (A) Body weight changes in mice over 14 days after intraperitoneal injection of AEVs at varying doses (50-800 mg/kg). Data are presented as mean ± SD (n = 8 per group). (B) Representative H&E-stained histological sections of major organs (heart, liver, spleen, lung, and kidney) from the high-dose group (800 mg/kg). Scale bar: 100 μm.


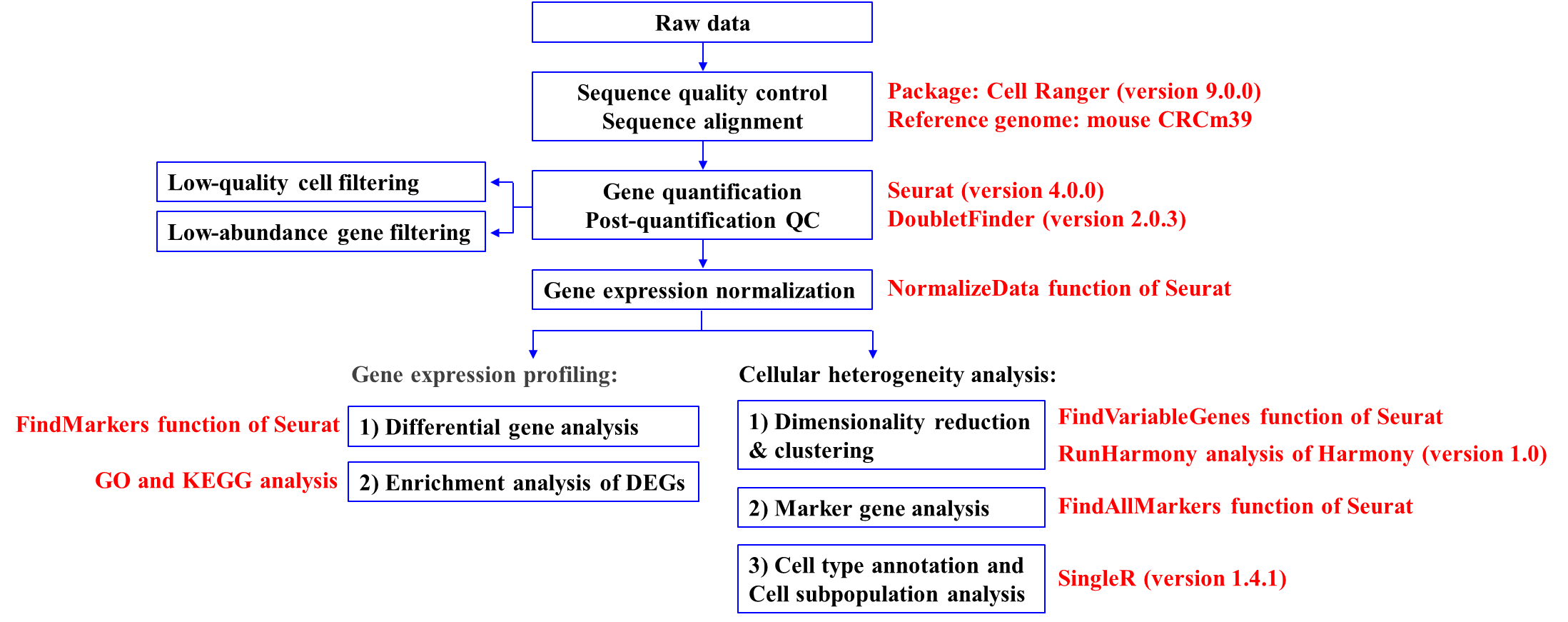


**Figure S8.** Workflow of single-cell RNA sequencing data analysis.


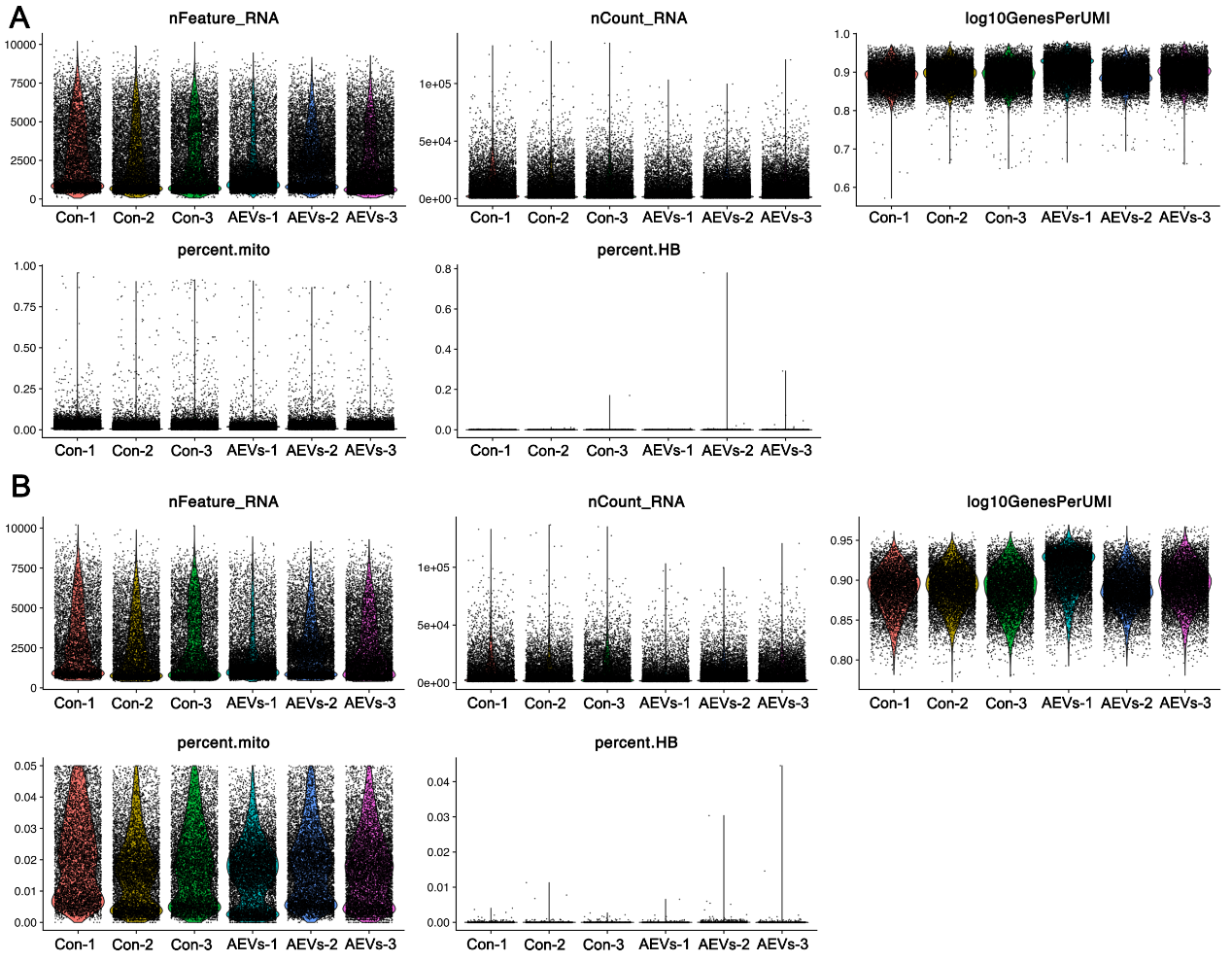


**Figure S9.** Quality control of scRNA-seq. Violin plots of scRNA-seq data quality control (A) and after quality control (B). nFeature_RNA: the number of genes; nCount_RNA: the number of UMIs; log10GenesPerUMI: the proportion of the number of genes in unit UMI; percent.mito: the percent of mitochondrial content; percent.HB: the proportion of red blood cell genes.


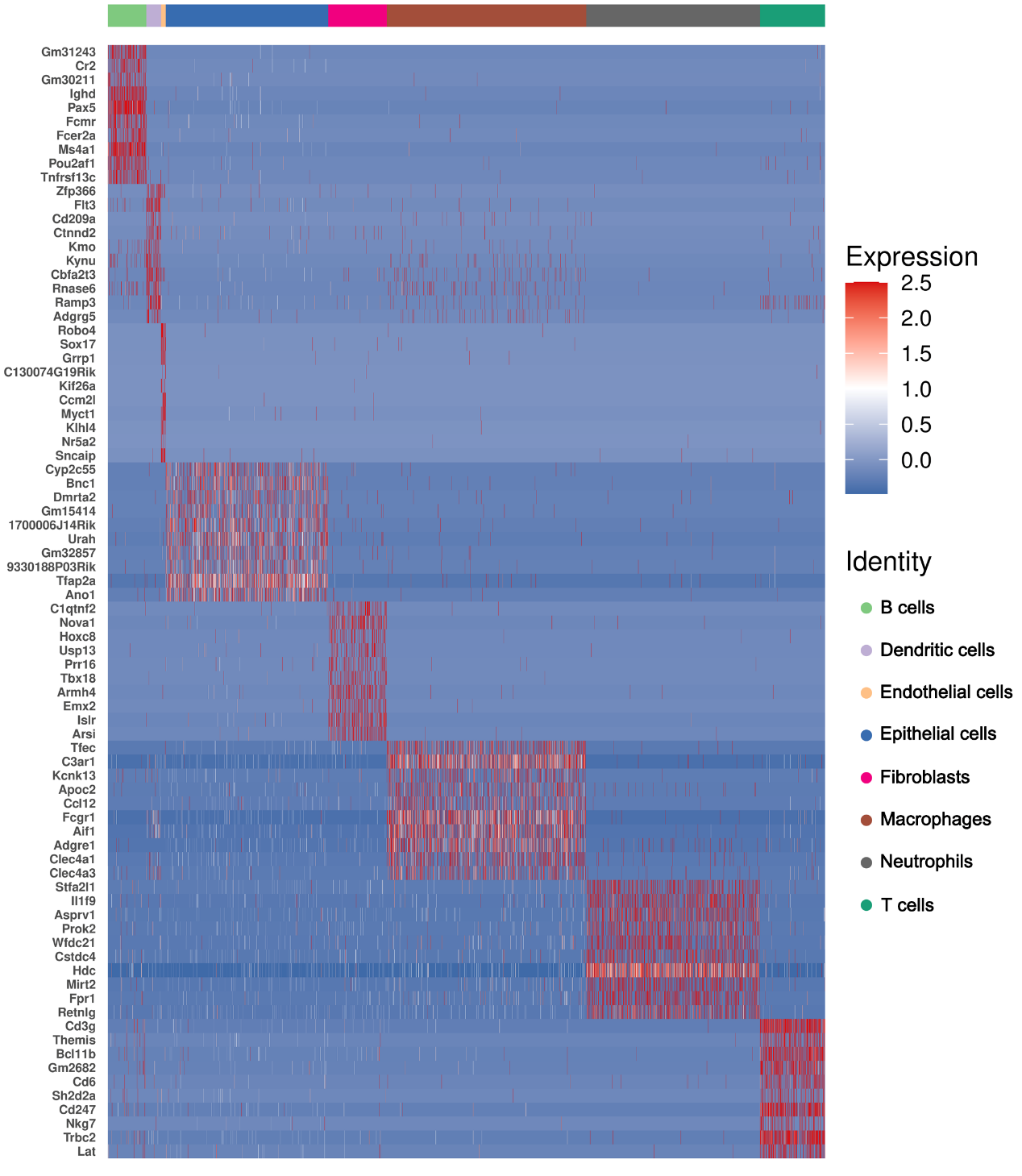


**Figure S10.** Heat map of top 10 marker gene expression in the different cell types identified by scRNA-seq.


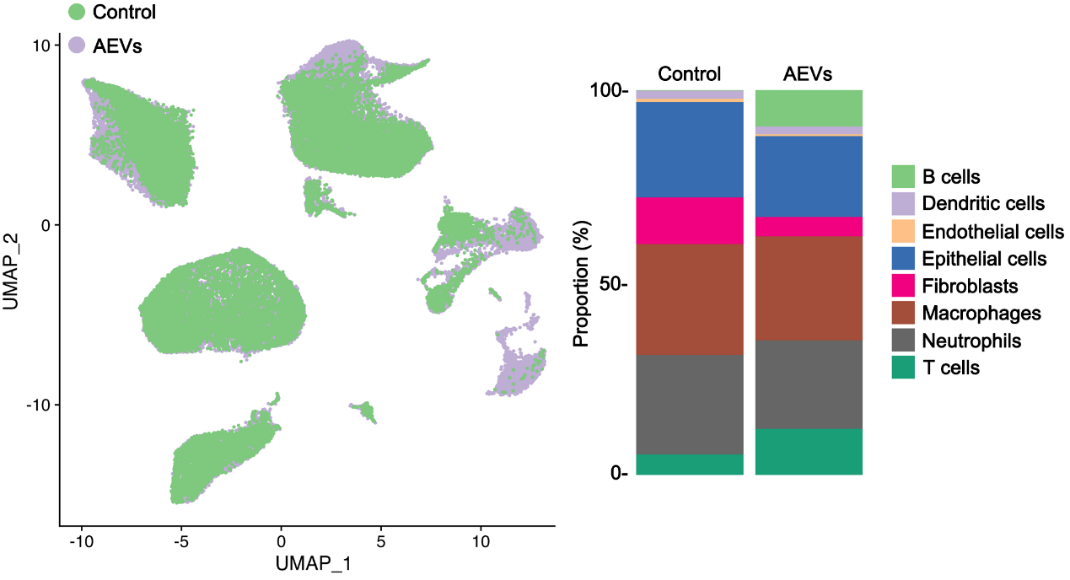


**Figure S11.** Changes of cells proportion before and after treatment of AEVs by scRNA-seq analysis.


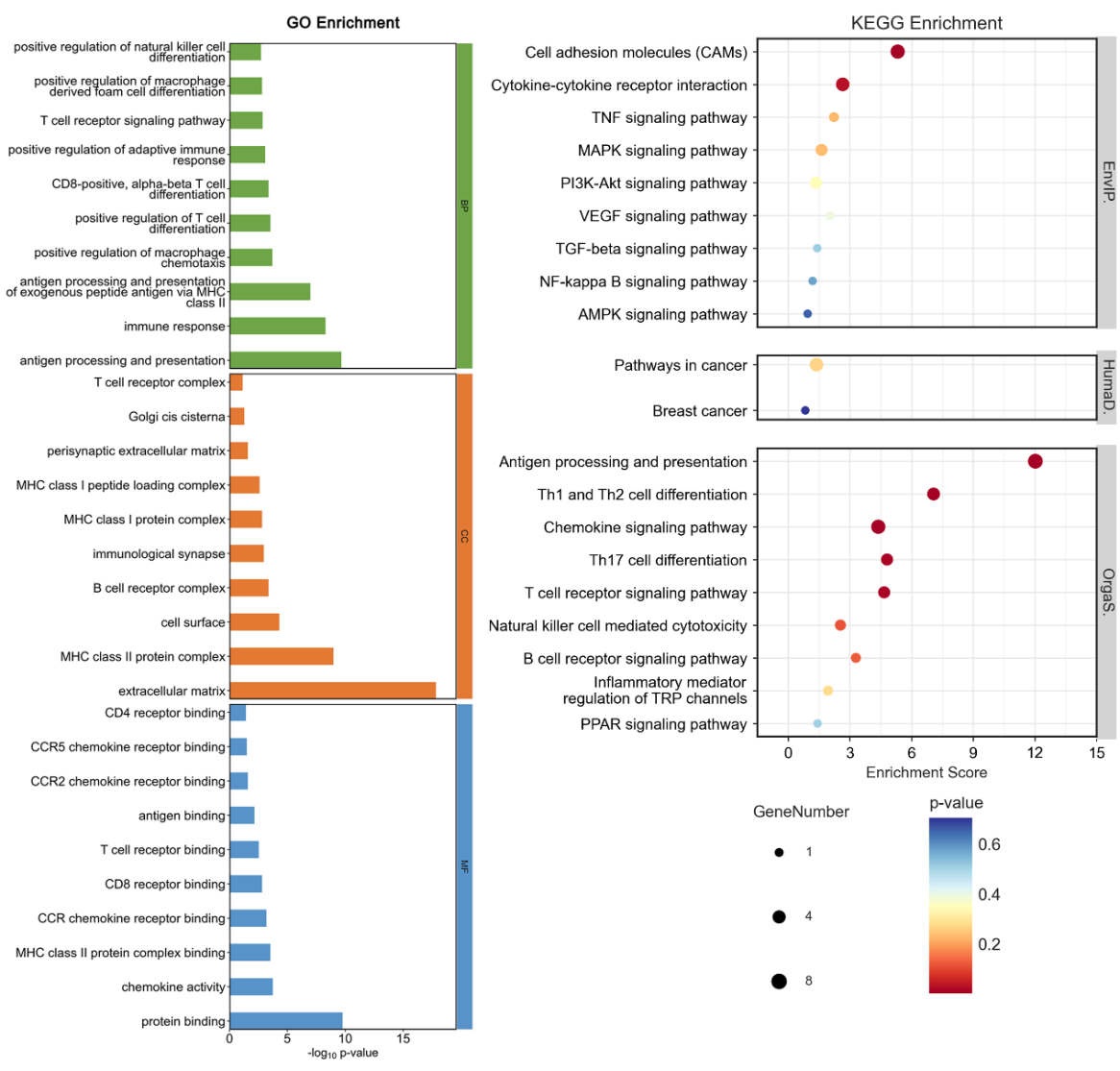


**Figure S12.** GO terms of the differentially expressed genes and KEGG pathway enrichment before and after treatment of AEVs by scRNA-seq analysis.


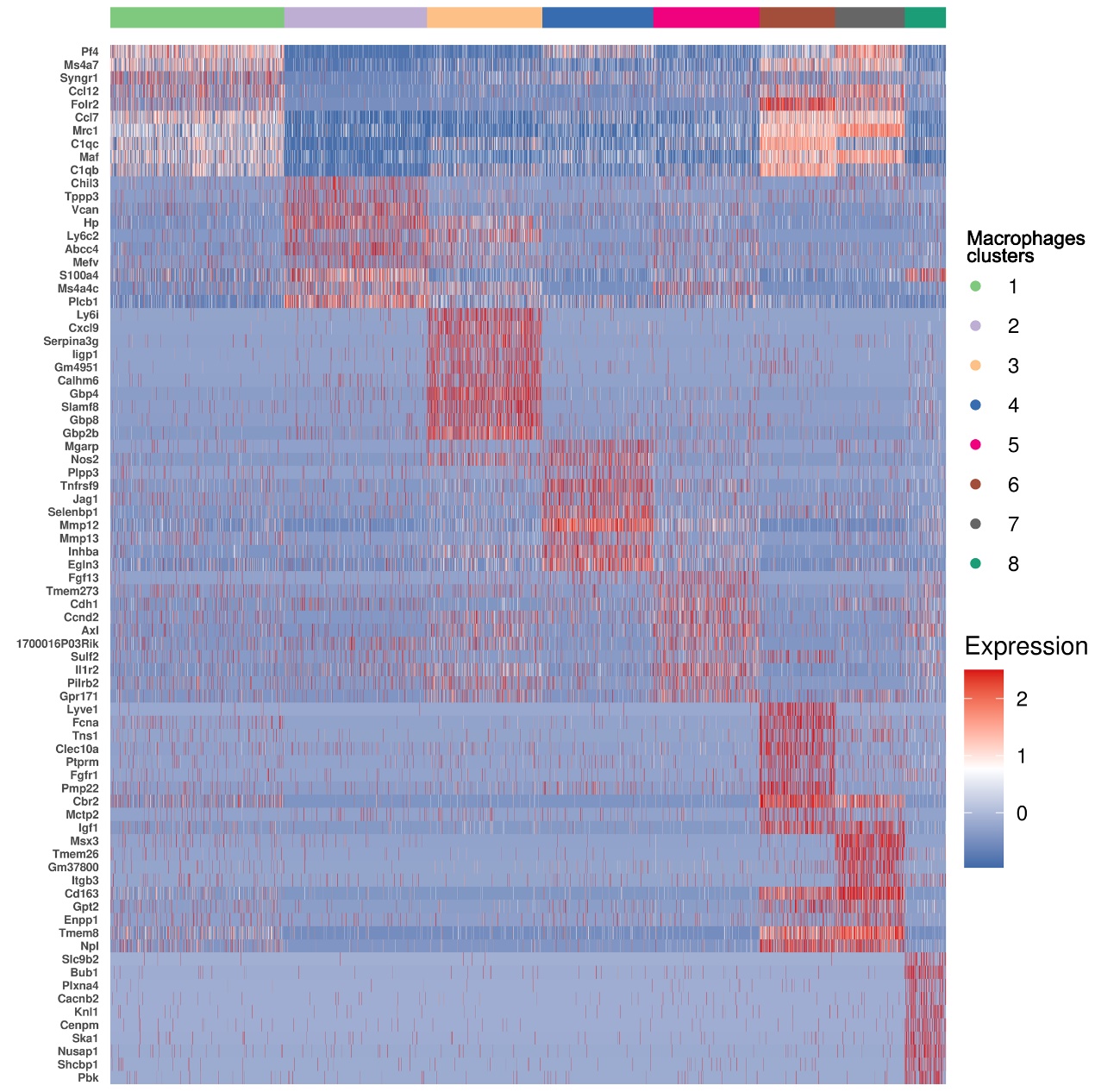


**Figure S13.** Heat map of top 10 marker gene expression in different macrophage clusters identified by scRNA-seq.


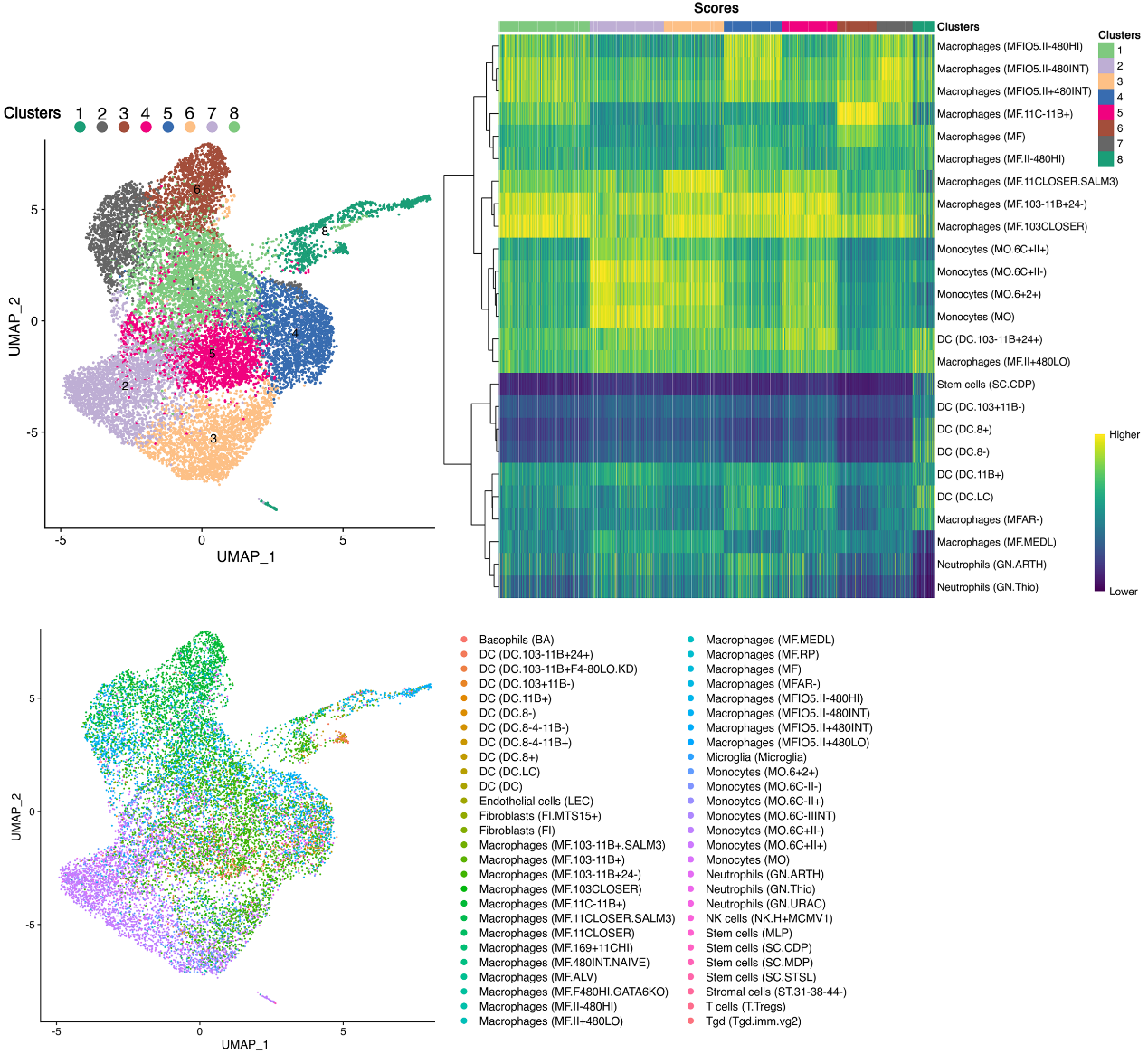


**Figure S14**. Cell typing of macrophages identified by scRNA-seq.


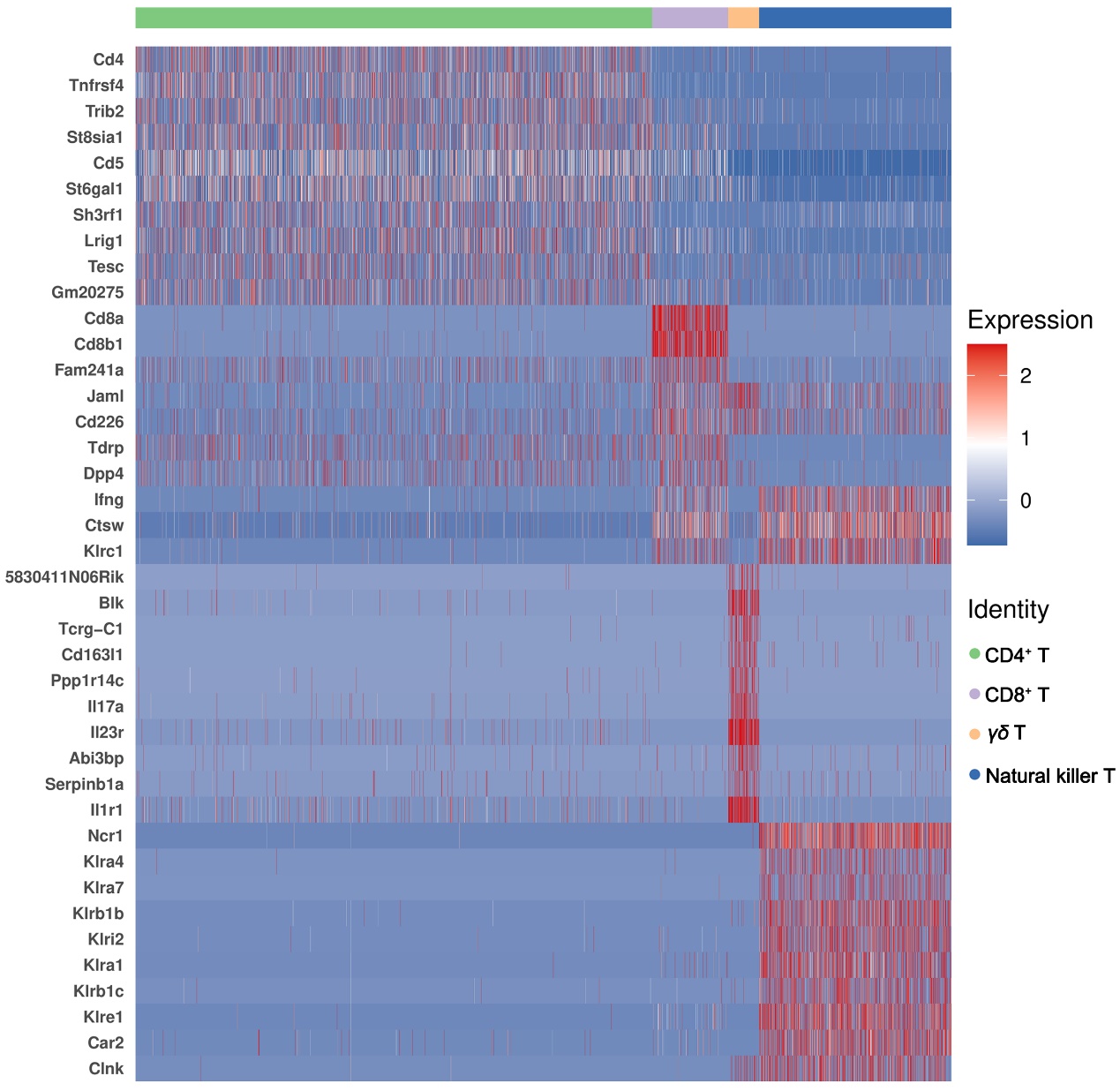


**Figure S15**. Heat map of top 10 marker gene expression in different T lymphocyte clusters identified by scRNA-seq.


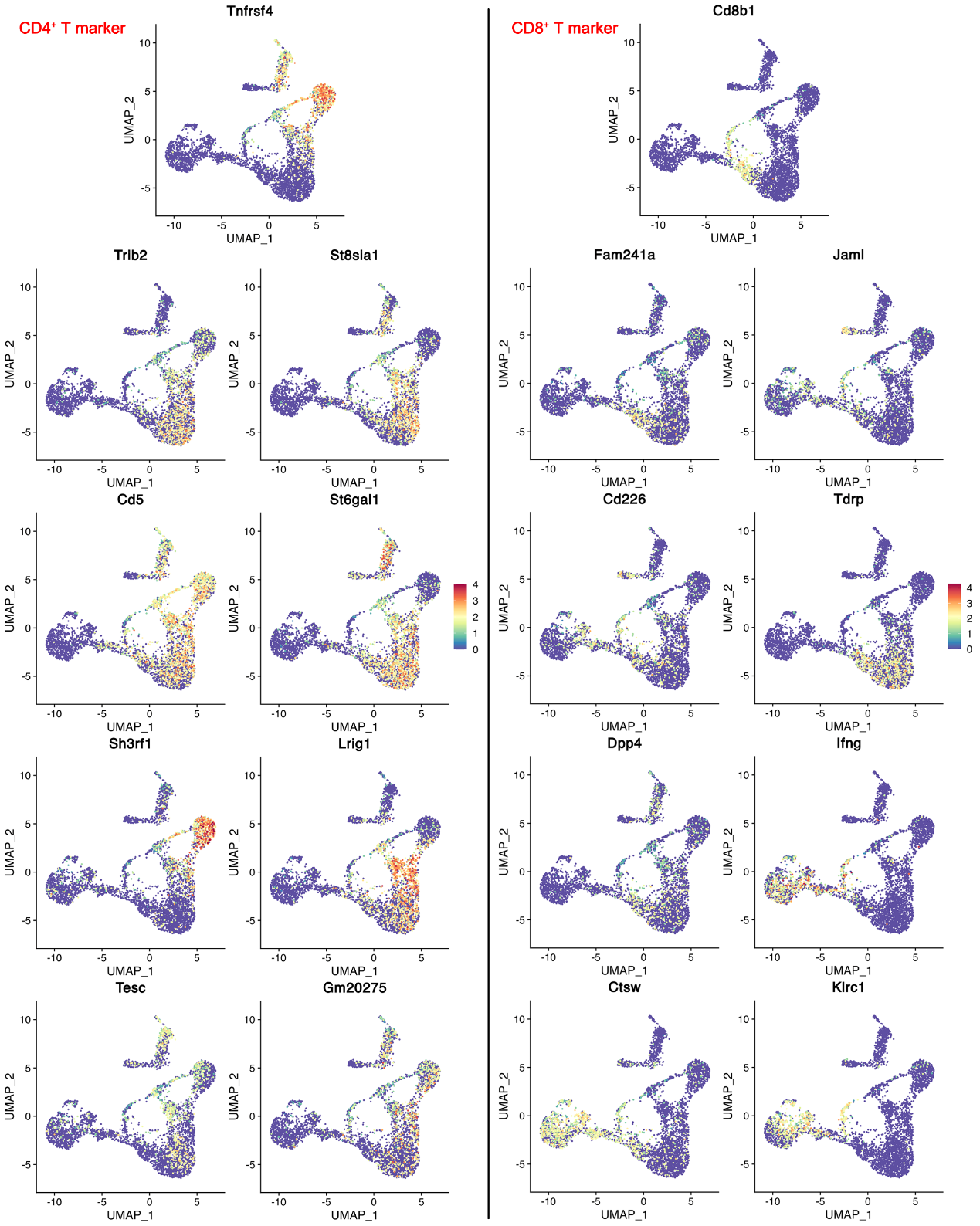


**Figure S16**. UMAP plots of expression for selected marker genes of CD4^+^ T and CD8^+^ T cells by scRNA-seq.


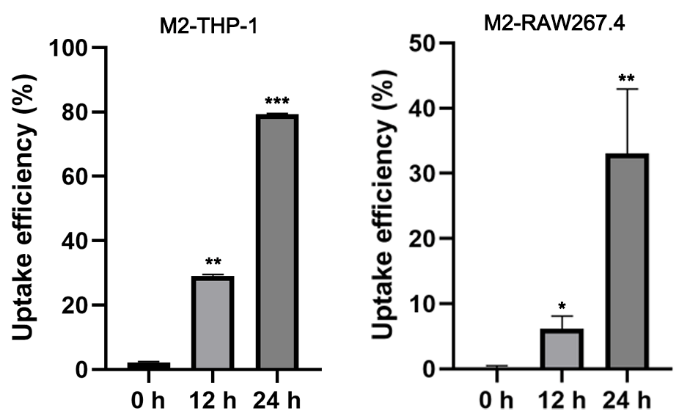


**Figure S17**. Uptake efficiency of AEVs quantified by flow cytometry. Data were mean ± SD (n = 3). ^*^*p* < 0.05, ^**^*p* < 0.01 and ^***^*p* < 0.001, compared to control.


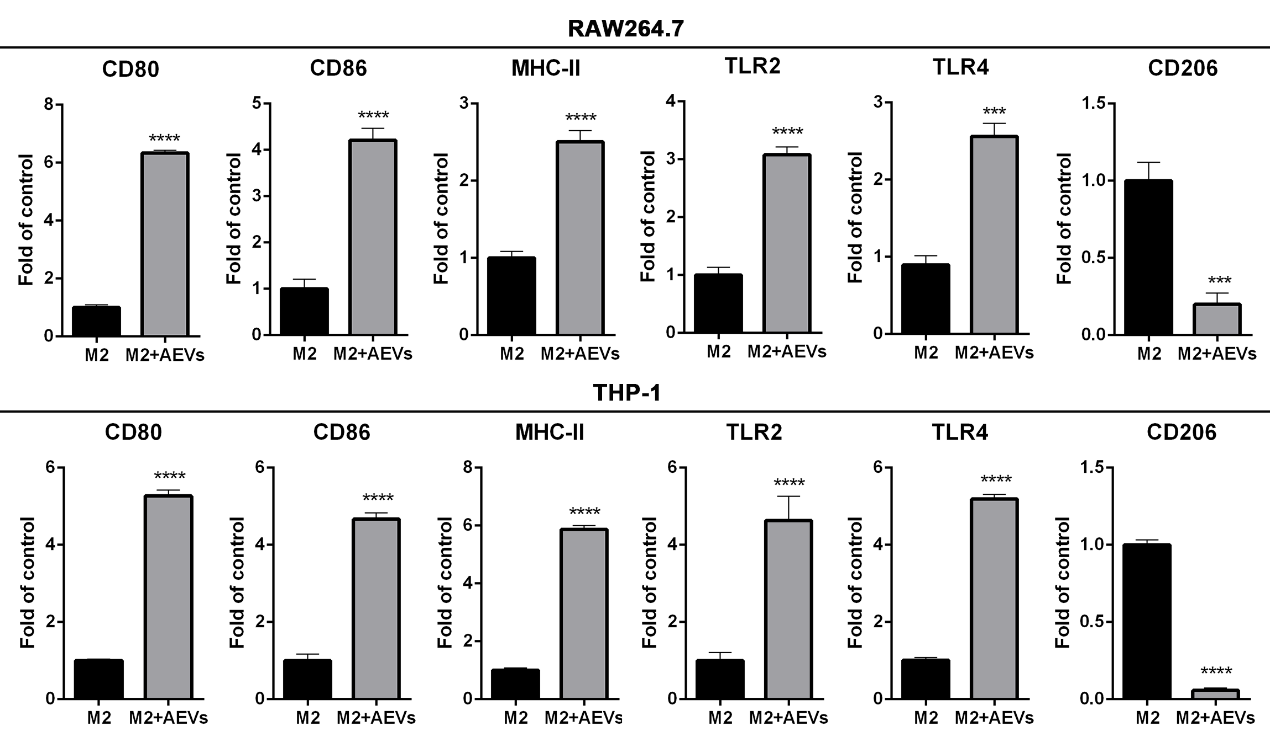


**Figure S18.** Quantitative analysis of marker expression after AEVs treatment. Data were mean ± SD (n = 3). ^***^*p* < 0.001, ^****^*p* < 0.0001 and compared to control.


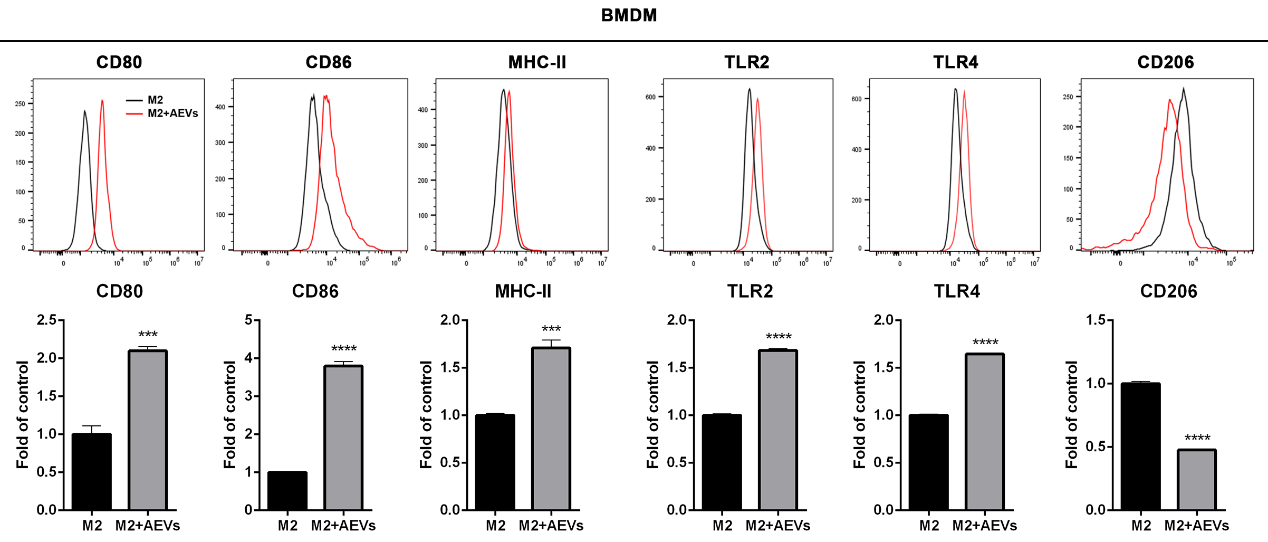


**Figure S19.** Surface marker expression profile of M2-polarized BMDMs treated with or without AEVs by flow cytometry. Data were mean ± SD (n = 3). ^***^*p* < 0.001, ^****^*p* < 0.0001 and compared to control.


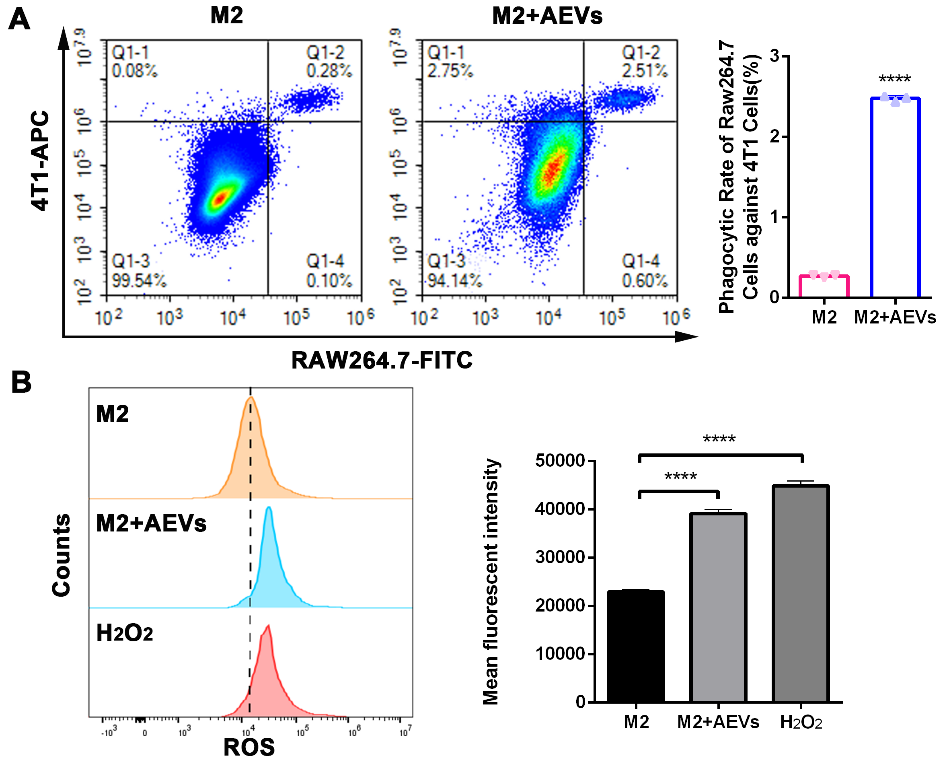


**Figure S20.** AEVs enhance phagocytic capacity and ROS production in macrophages. (A) Phagocytosis assay showing fold-change in macrophage uptake after AEV treatment compared to untreated controls. (B) ROS generation measured by DCFH-DA fluorescence in AEV-treated versus control macrophages. Data represent mean ± SD (n = 3). ^****^*p* < 0.0001 and compared to control.


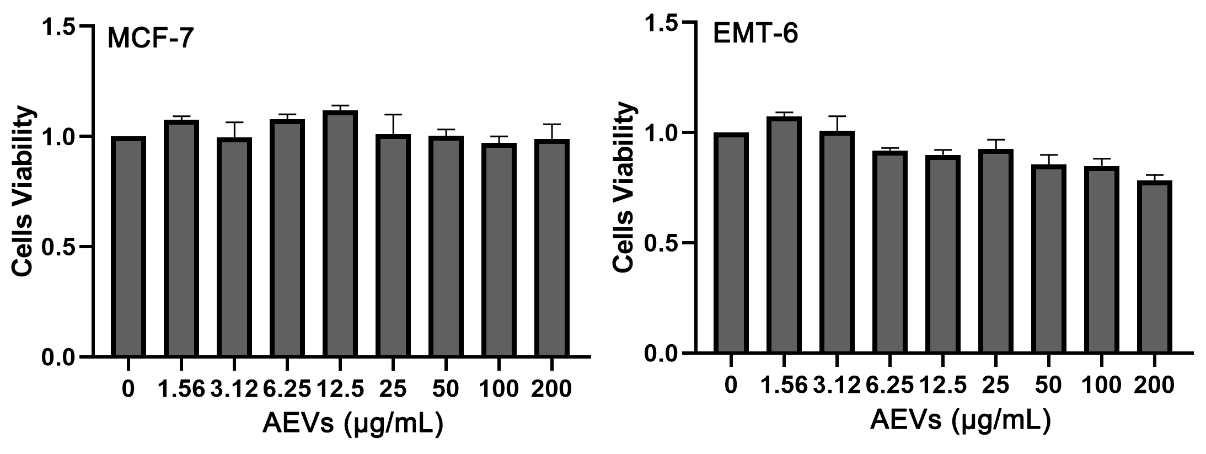


**Figure S21.** Cells viability of AEVs treatment relative to the control determined by MTT assay. Data were the mean ± SD (n = 3).


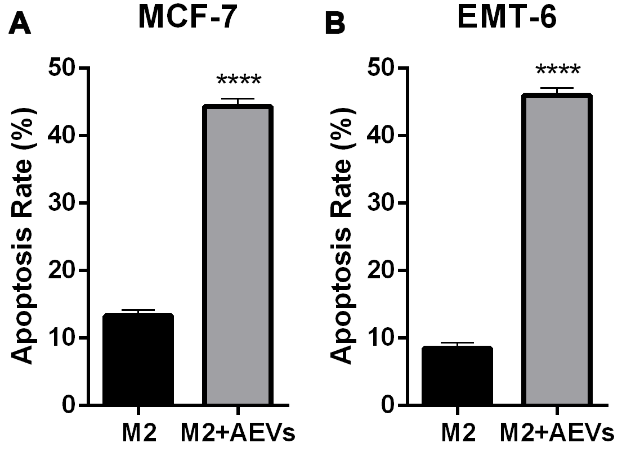


**Figure S22.** Quantitative analysis of apoptosis after treatment with AEVs. (A) MCF-7. Data were mean ± SD (n = 3). ^****^*p* < 0.0001 and compared to control.


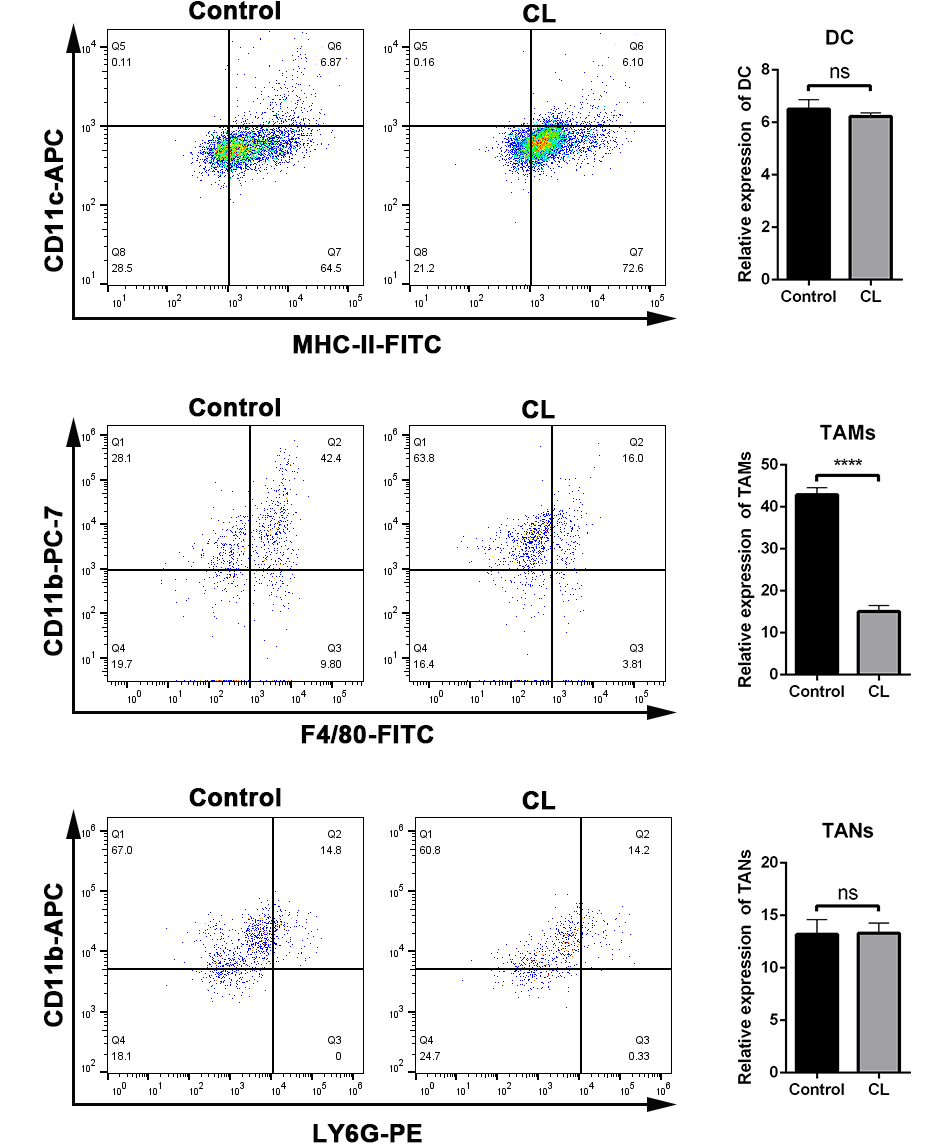


**Figure S23.** CL treatment specifically depletes tumor-associated macrophages (TAMs) without affecting other myeloid populations. Data are presented as mean ± SD; ^****^*p* < 0.0001; NS, not significant (p > 0.05).


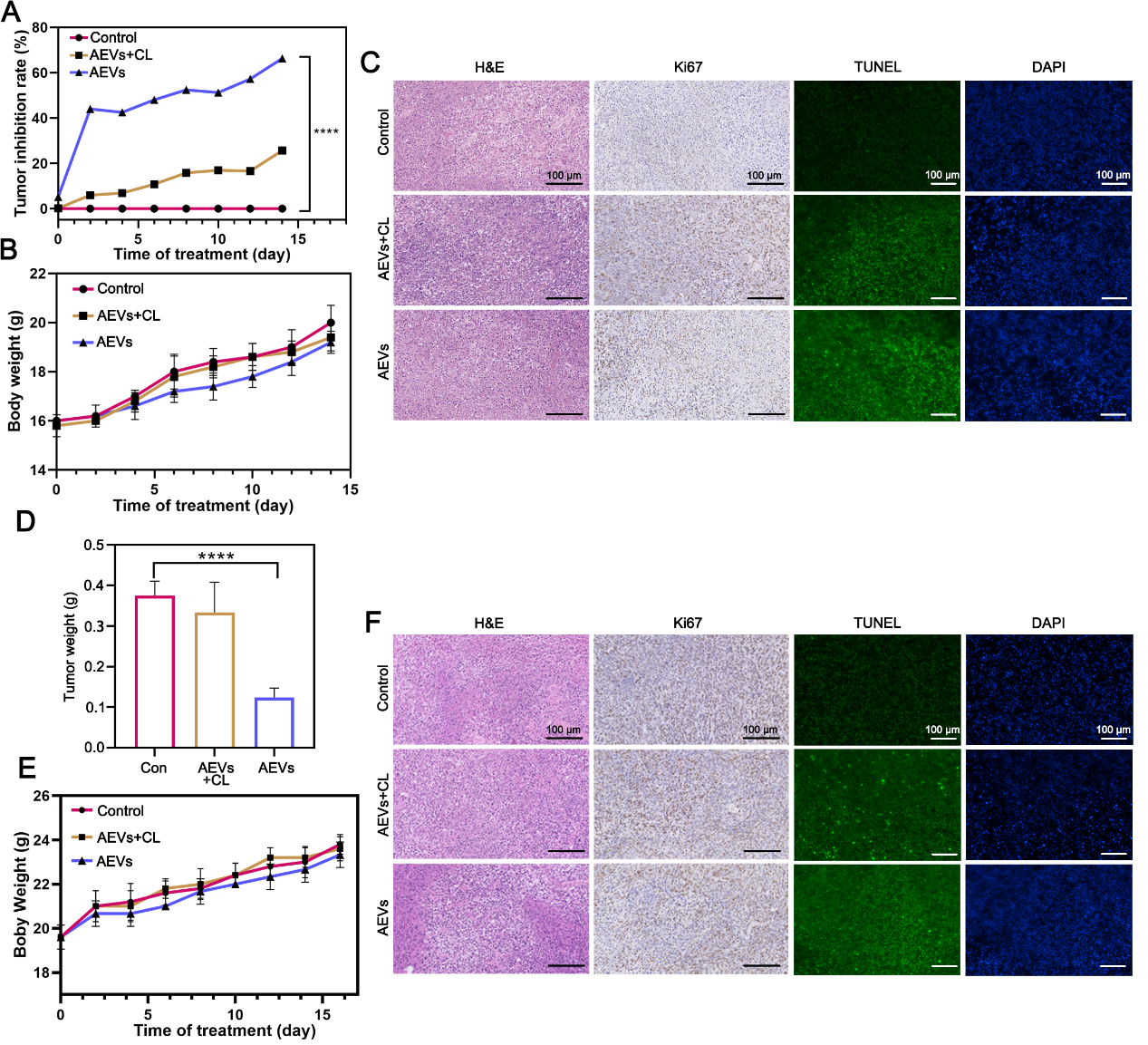


**Figure S24.** Tumor inhibition rate (A), body weight (B), and H&E, Ki67, TUNEL staining images (C) of tumor slices after administration at day 14 in EMT-6 tumor-bearing mice model. Tumor weight (D), body weight (E), and H&E, Ki67, TUNEL staining images (F) of tumor slices after administration at day 16 in C127 tumor-bearing mice model. Data were mean ± SD (n = 5). ^****^*p* < 0.0001.


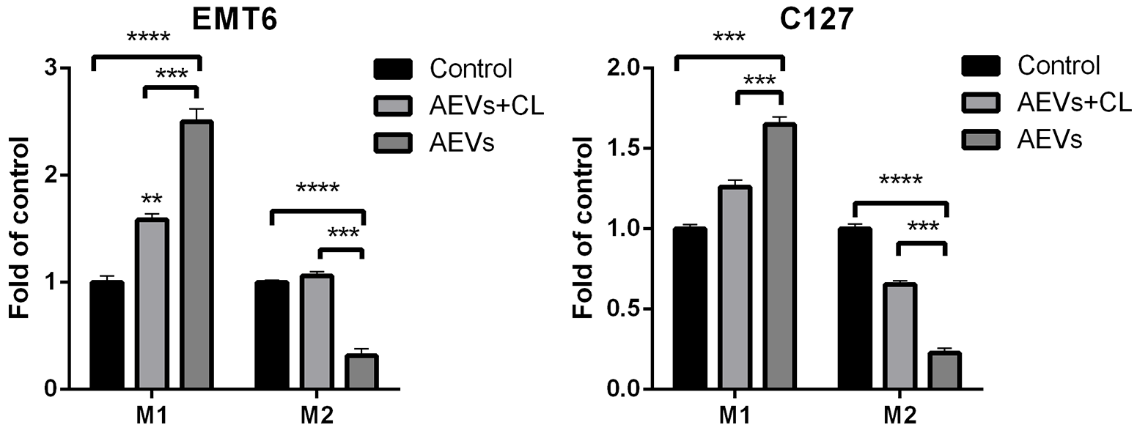


**Figure S25.** Quantification of M1-polarized cells and M2-polarized cells. Data were mean ± SD (n = 3). ^****^*p* < 0.0001 and compared to control.


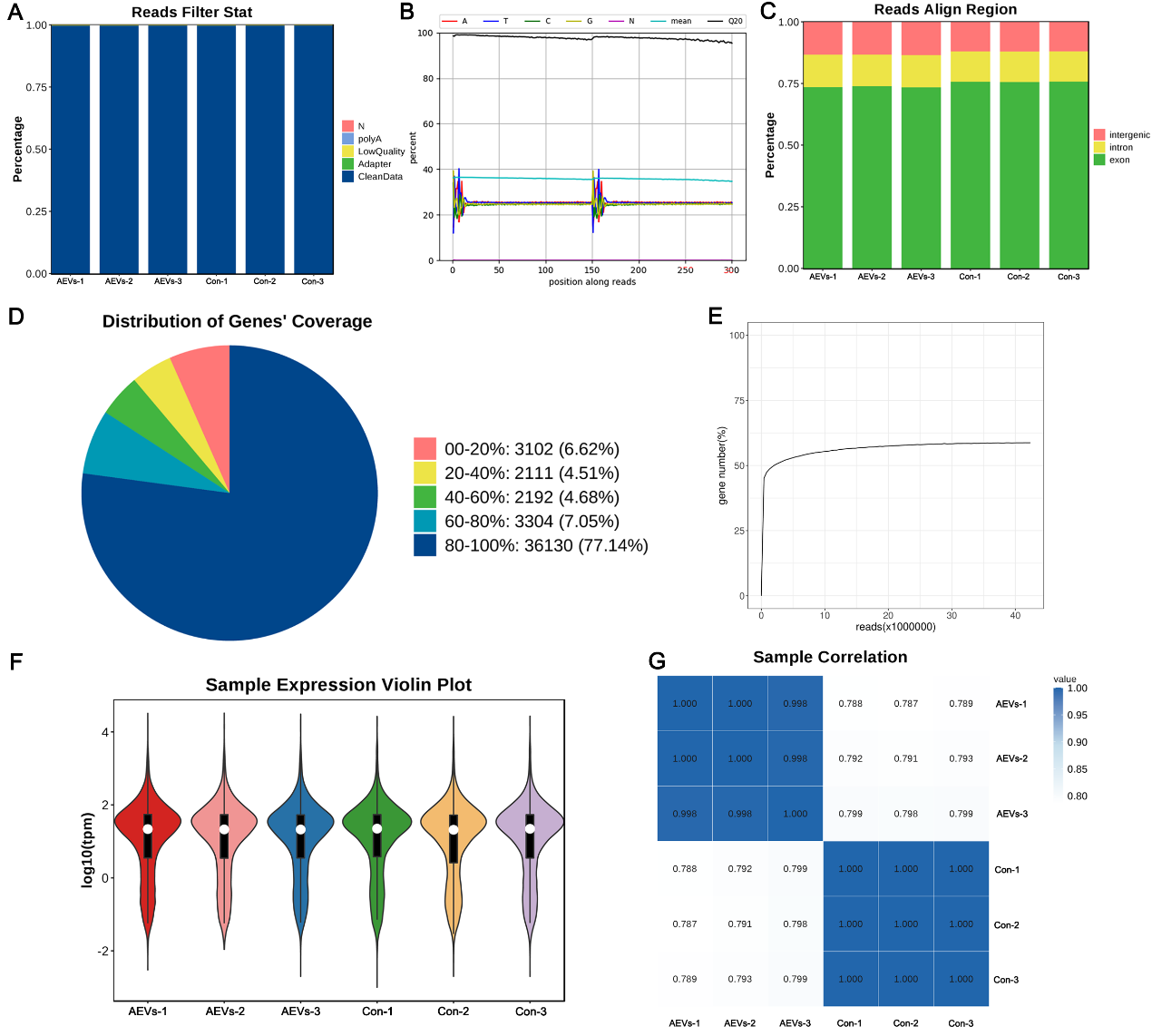


**Figure S26.** Quality control of mRNA-seq of M2-RAW264.7 cells after treatment of AEVs. (A) Data preprocessing distribution (%) after filtering low-quality data. (B) Representative base distribution. (C) The statistical map of the reference area. (D) Distribution of genes’ coverage. (E) Distribution of sequencing saturation. (F) Violin map of gene expression volume. (G) Heat map of sample correlation.


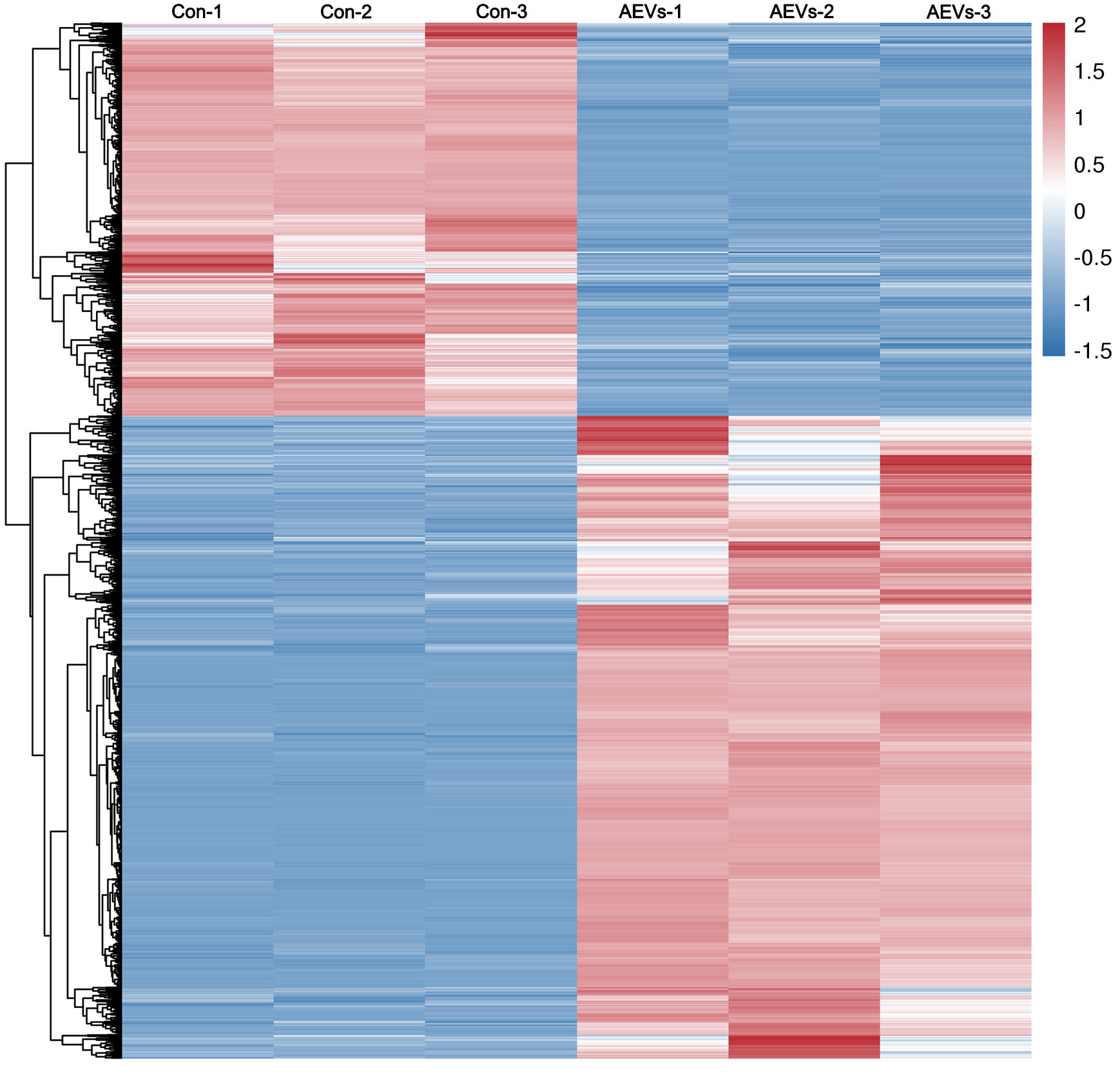


**Figure S27**. Heat map of mRNA expression in M2-RAW264.7 cells before and after treatment of AEVs in mRNA-seq analysis.


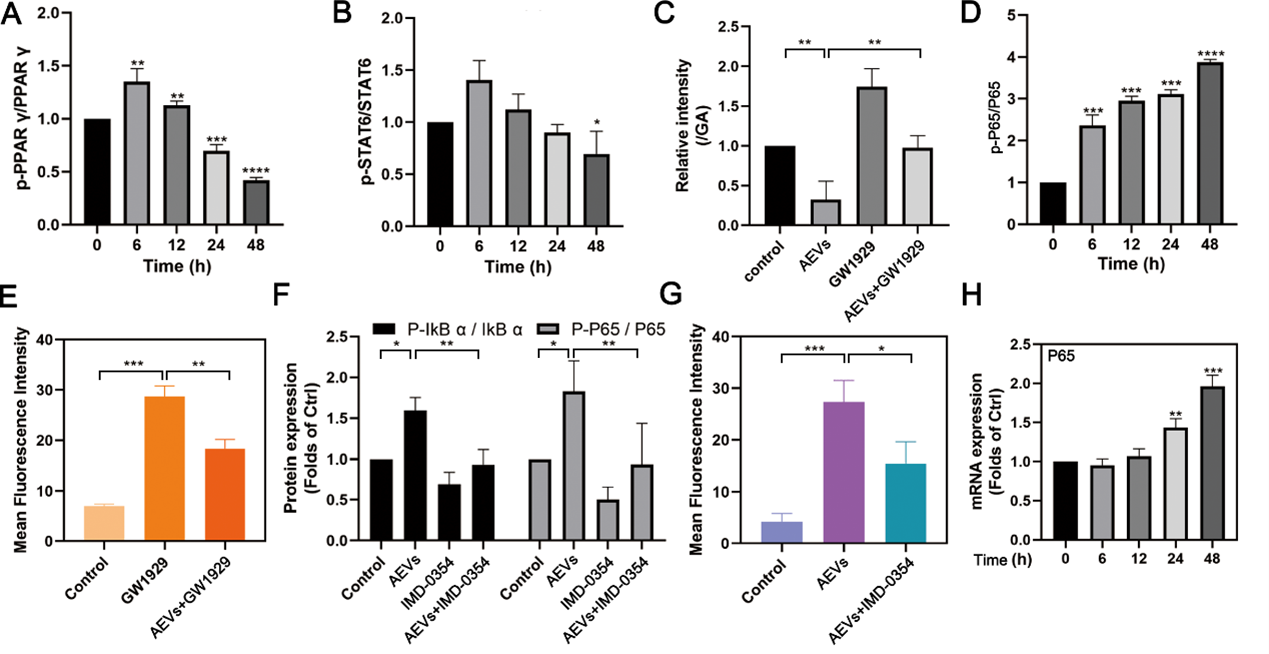


**Figure S28** The levels of p-PPARγ/PPARγ (A), p-STAT6/STAT6 (B), PPARγ (C), p-P65/P65 (D), and p-IκBα/IκBα (F) of M2-RAW264.7 cells treated with various formulations for different time by Western blotting. (E,G) Mean fluorescence intensity of M2-RAW264.7 cells treated with various formulations by using a high-content screening system. (H) Relative mRNA levels of P65 in M2-RAW264.7 cells after treatment with AEVs determined by qRT-PCR analysis. Data were mean ± SD (n = 3). ^*^*p* < 0.05, ^**^*p* < 0.01, ^***^*p* < 0.001, and ^****^*p* < 0.0001, compared to control.


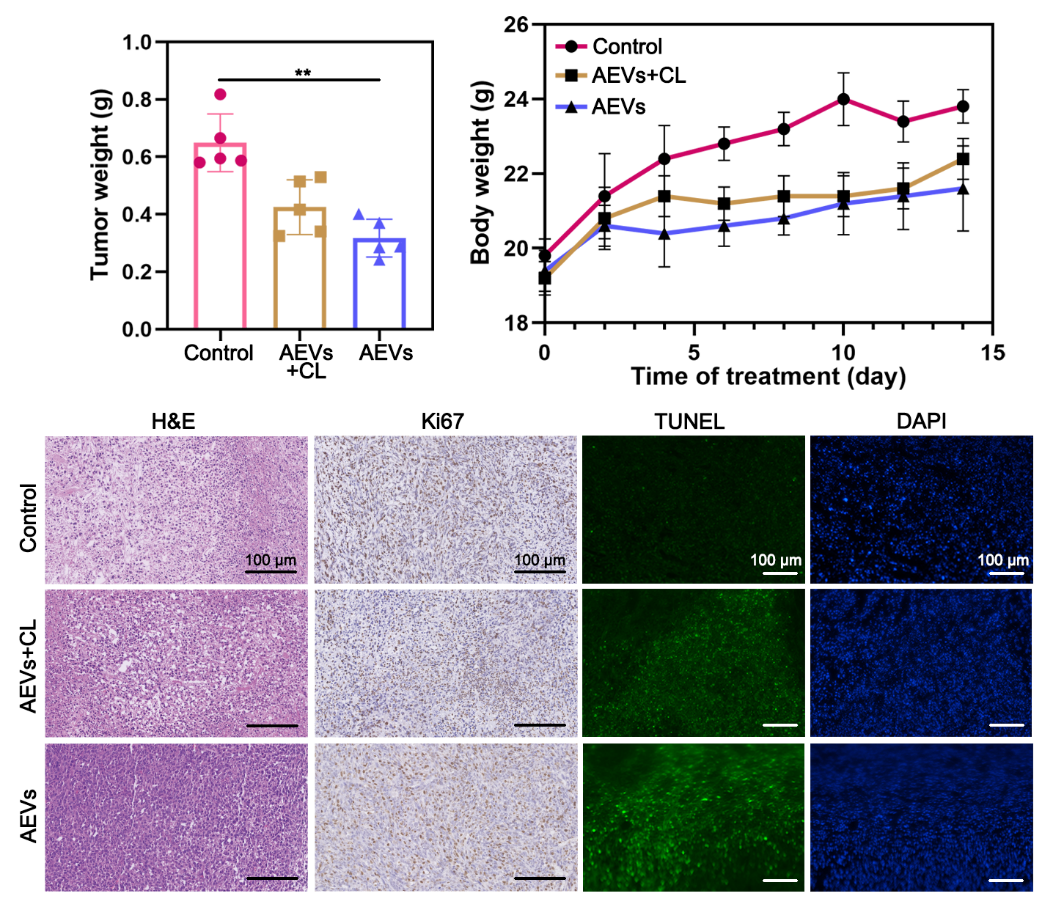


**Figure S29.** Tumor weight, body weight, and H&E, Ki67, TUNEL staining images of tumor slices after administration at day 14 in 4T1 tumor-bearing mice model. Data were mean ± SD (n = 5). ^**^*p* < 0.01.


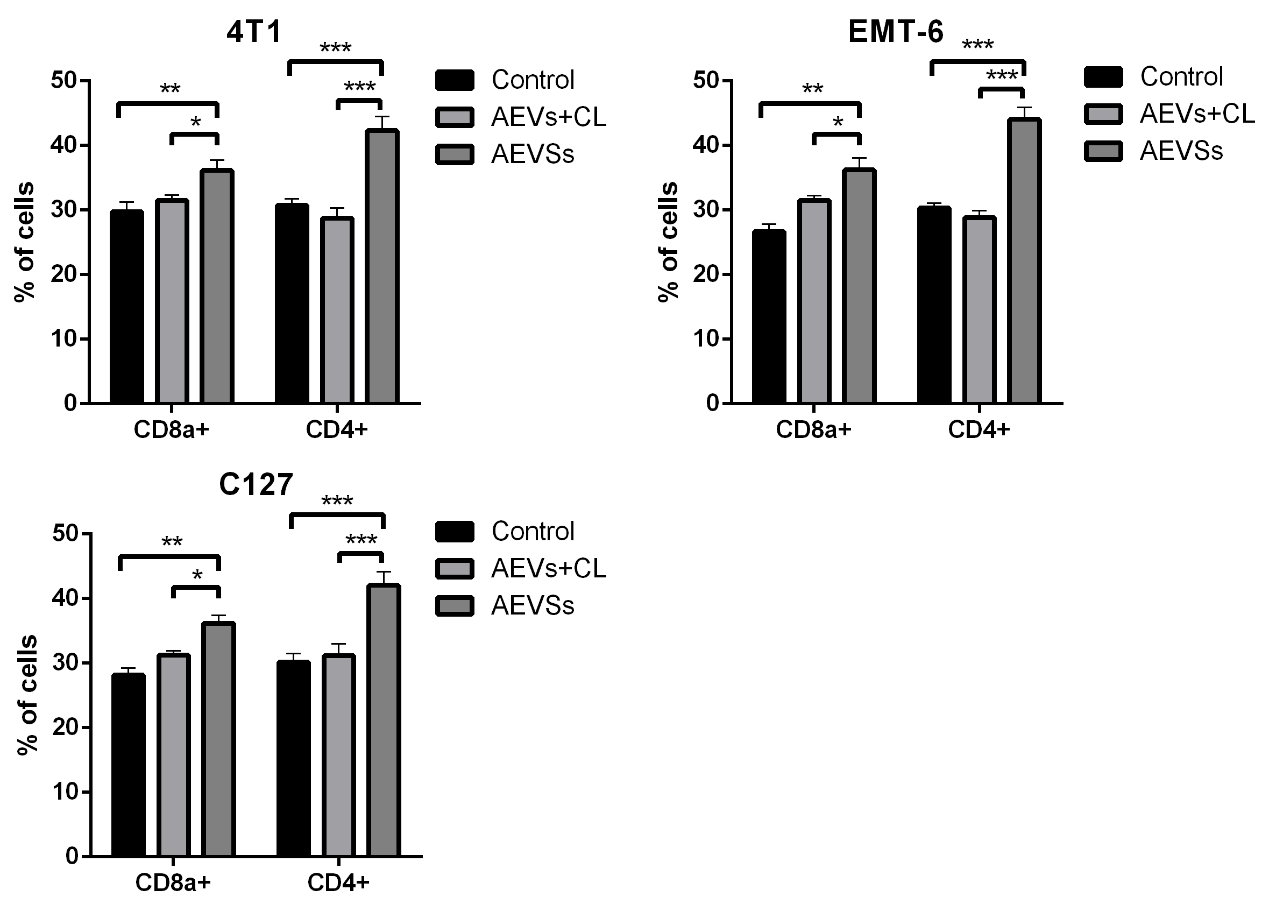
**Figure S30.** Quantification of CD8a^+^ T cells and CD4^+^ T cells as a percentage of total CD3^+^ T cells. Data were mean ± SD (n = 3). ^*^*p* < 0.05, ^**^*p* < 0.01, and ^***^*p* < 0.001, compared to control.
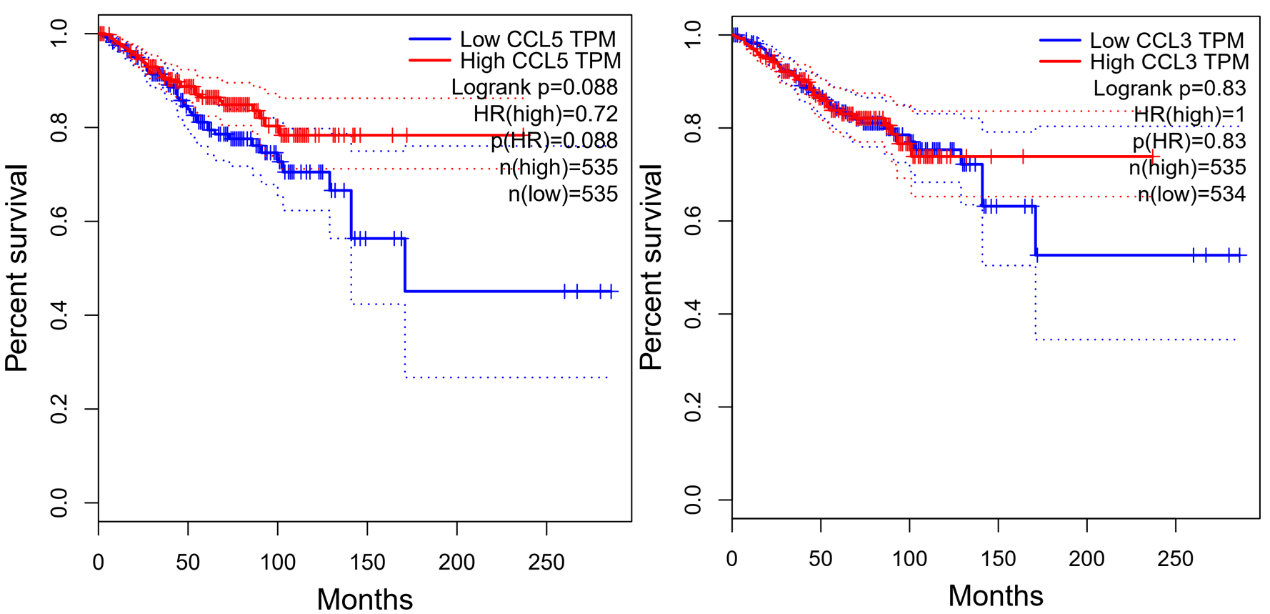


**Figure S31.** Disease free survival analysis of CCL5 and CCL3 expression in breast cancer patients from GEPIA database (<http://gepia.cancer-pku.cn/>).


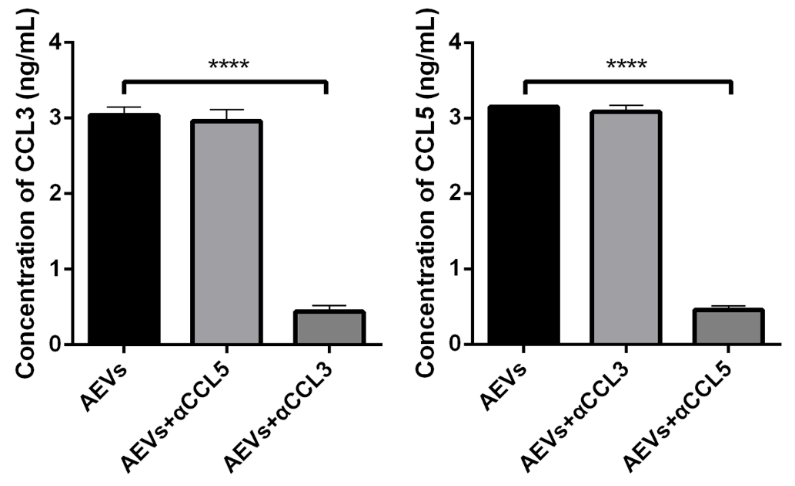


**Figure S32.** Specificity validation of CCL3 and CCL5 neutralizing antibodies by ELISA. Data are presented as mean ± SD (n = 3). ^****^*p* < 0.0001.
